# A multi-centre investigation of delivering national guidelines on exercise training for men with advanced prostate cancer undergoing androgen deprivation therapy in the UK NHS

**DOI:** 10.1371/journal.pone.0197606

**Published:** 2018-07-05

**Authors:** Liam Bourke, Rebecca Turner, Rosa Greasley, Eileen Sutton, Liz Steed, Dianna Smith, Janet Brown, Ben Kelly, Claire Hulme, Diana Greenfield, Raj Persad, Amanda Farrin, Jenny Hewison, Derek J. Rosario, on behalf of the STAMINA investigators

**Affiliations:** 1 Health and Wellbeing, Sheffield Hallam University, Sheffield United Kingdom; 2 School of Social and Community Medicine, University of Bristol, Bristol United Kingdom; 3 Centre for Primary Care and Public Health, Queen Mary University of London, London United Kingdom; 4 Department of Geography and Environment, University of Southampton, Southampton United Kingdom; 5 Department of Oncology and Metabolism, University of Sheffield, Sheffield United Kingdom; 6 Nuffield Health Research Group, Epsom Gateway, Epsom, Surrey United Kingdom; 7 Leeds Institute of Health Sciences, Faculty of Medicine and Health, University of Leeds, Leeds United Kingdom; 8 Sheffield Teaching Hospitals NHS Foundation Trust, Sheffield United Kingdom; 9 Department of Urology/Surgery, Southmead Hospital, Bristol, United Kingdom; Thomas Jefferson University, UNITED STATES

## Abstract

**Background:**

National guidelines (NICE-CG175) recommended 12 weeks of supervised exercise training for men treated with androgen deprivation therapy (ADT) for prostate cancer to counter debilitating adverse effects of castration. As with other chronic conditions where exercise is indicated, it is uncertain if these services are being delivered in the health services. The aim of this multi-centre investigation was to examine what exercise referral is currently available for men on ADT as provided by the NHS and if a supervised, individually-tailored exercise training package (as per the national NICE guidelines CG175) is embedded within prostate cancer care.

**Methods:**

A multi-centre investigation of current National Health Service (NHS) care involving a web-based survey of NHS prostate cancer care, five focus groups involving 26 men on ADT and 37 semi-structured interviews with healthcare professionals (HCPs) involved in the management of prostate cancer. Descriptive statistics and thematic analysis evaluated quantitative and qualitative data, respectively. Qualitative methods followed COREQ standards.

**Results:**

HCPs and men on ADT asserted that medical castration has a serious and debilitating impact on many features of men's quality of life. There is support for exercise training programmes as part of cancer care and patients would support their initiation soon after diagnosis. Involving the Multidisciplinary Team (MDT) is proposed as key to this. Critically, traditional values in oncology would need to be overcome for widespread acceptance. Specialist further training for HCPs around behaviour change support could encourage this. Given that these schemes are seen as a fundamental part of cancer care, it is felt the NHS should commission and support provision. 79 representatives of 154 NHS trusts (51%) provided survey data on current delivery: only 17% could provide supervised exercise as per CG175.

**Conclusions:**

Evidence-based national exercise guidelines are not being delivered to men on ADT as intended. Traditional values in oncology and the need for NHS financial support are seen as major barriers to provision of current best practice guidelines. Despite this both HCPs and men on ADT are in favour of such programmes being a fundamental part of their cancer care.

## Introduction

In 2014, approximately 47,000 men were newly diagnosed with prostate cancer in the UK, with 11,000 resultant deaths per annum.[1] Androgen deprivation therapy (ADT)[2] is standard treatment in the NHS[3] delivered neo-adjuvantly with radiotherapy for localised and locally advanced disease and as monotherapy for metastatic disease (more recently in combination with taxane-based chemotherapy).[4] Around one half of men with prostate cancer will undergo such treatment.[5] Given current incidence data for prostate cancer it is likely that around 20,000 men are initiated on ADT annually and approximately 125,000 men in the UK will be maintained on ADT this year. It is estimated that by 2020 and 2030 this will be over 200,000 and 300,000 men respectively.[6]

ADT adversely impacts quality of life (QoL) causing fatigue, reduced muscle mass, sexual problems, and increases risk of fracture and acute kidney injury.[5,7–14] In men with advanced prostate cancer the prevalence of dementia, depression, anxiety or psychosis is around 1 in 4.[15] Furthermore, long-term ADT has been linked with worsening cardiovascular health.[16] This is of concern as men with prostate cancer already constitute a high-risk population for cardiovascular disease.[17] As well as the distress and loss of QoL to the individual, these side-effects place additional burden on healthcare services and support networks.

Unmet needs and ongoing support requirements from men diagnosed with prostate cancer are established.[18,19] Numerous strategies for managing side-effects are tried in practice, but exercise training is the only evidence-based supportive therapy for improving disease-specific QoL in men on ADT [20,21]. The National Institute for Health and Care Excellence (NICE) in the UK (NICE-CG175) and the European Association of Urology (EAU) have both recommended supervised exercise training as part of standard treatment for men with prostate cancer on long-term ADT.

The aim of this multi-centre investigation was to examine what exercise referral is currently available for men on ADT as provided by the NHS and if a supervised, individually-tailored exercise training package (as per the national NICE guidelines CG175) is embedded within prostate cancer care.

## Materials and methods

UK NHS (15/SW/0260) and University ethical boards provided approval for this study. The programme of work consisted of 3 components:, semi-structured interviews with healthcare professionals (HCPs), focus groups with men with prostate cancer on ADT and a national online survey of members of stakeholder organisations involved in the delivery of prostate cancer care.

Methods for qualitative reporting were guided by the COREQ standards [22] (S1 File). Interview and focus group participants were recruited between August 2015 and June 2016: written informed consent was obtained from participants.

### Semi-structured interviews

Semi-structured qualitative interviews were conducted with HCPs recruited via professional organisations and local authority employees working in diverse roles representing different disciplines within the NHS prostate cancer care and exercise referral pathway (see Table 1). Interviews (n = 37) were conducted either face-to-face (n = 7) or by telephone (n = 30) and lasted between 20 and 50 minutes. Interviews covered *inter alia* HCP roles, adverse effects of ADT, awareness, and practice with respect to the recent national (NICE-CG175) guidelines, problems with offering structured exercise programmes in standard care and the need for further education/training of HCPs (S2 File).

**Table 1:** Characteristics of healthcare professionals participating in interviews.

**Profession**	Consultant Urologist	24.3% (9)
	Oncologist	27% (10)
	Clinical Nurse Specialist	16.2% (6)
	General Practitioner	8.1% (3)
	Physiotherapist	8.1% (3)
	Exercise Specialist	5.4% (2)
	Service Manager	2.7% (1)
	Clinical Commissioner	8.1% (3)
	Primary Care Physician	2.7% (1)
**Institution**	Teaching Hospital	24.3% (9)
	District Hospital	18.9% (7)
	University	2.7% (1)
	Community	13.5% (5)
	Cancer centre	29.7% (11)
	Primary Care	10.8% (4)

### Focus groups

Focus groups were facilitated by members of the research team supported by a patient representative. Twenty-six men with prostate cancer on ADT for at least 6 months were recruited from urology out-patients departments at the Royal Hallamshire and Chesterfield Royal Hospitals (approached by the research team during routine follow-up, from prostate cancer support groups, or via recruitment posters). See Table 2 for details. Study information was posted to the homes of men who expressed an interest, and phone calls were made after 24 hours of them receiving this information to invite them to a group. Participants completed a demographic questionnaire, the Godin Leisure-Time Exercise Questionnaire[23] and WHODAS II Questionnaire[24],upon arrival.

**Table 2:** Focus group characteristics.

**Age (yrs)**	55–59	7.7% (2)
	60–69	38.5% (10)
	70–79	38.5% (10)
	80–89	15.4% (4)
**Ethnicity**	White British	96.2% (25)
	African/ Afro-Caribbean origin	3.8% (1)
**Educational status**	Degree or other higher education	26.9% (7)
	‘A’ level or equivalent in other country	14.3% (4)
	GSCE or equivalent in other country	7.7% (2)
	Other qualification	10.7% (3)
	No formal qualification	35.7% (10)
**Marital Status**	Married or living with partner	80.8% (21)
	Divorced or separated	11.5% (3)
	Widowed	7.7% (2)
**Employment status**	Full time employment	7.7% (2)
	Self-employment	7.7% (2)
	Retired	64.3% (18)
	Voluntary Work	3.8% (1)
	Sick or disabled	3.8% (1)
	Other	7.7% (2)
**Length of diagnosis**	<6 months	7.7% (2)
	6 months– 3 years	50.0% (13)
	3–5 years	15.4% (4)
	5+ years	26.9% (7)
**Length of ADT**	<6 months	7.7% (2)
	6 months– 3 years	65.4% (17)
	3–5 years	15.4% (4)
	5+ years Need to add up to 26 or add in ‘missing’	11.5% (3)
Co-morbidities reported*	High Blood pressure	7
	Type 2 diabetes	3
	Respiratory	6
	Cardiac	4
	Arthritis	1
	Colitis	3
	Other	4
	Musculoskeletal/ spinal	3
	None	6

*11 men reported more than one co-morbidity

During the focus groups (S3 File) discussions firstly centred around physical activity and experiences of prostate cancer, followed by more in-depth questions regarding QoL and views on exercise training as a way to manage side-effects of ADT. The practicalities of accessing an integrated exercise programme were also debated. Adverse effects of ADT and coping styles were discussed within the groups to understand how exercise might be embedded in standard care. All focus groups and interview participants provided informed consent.

### Electronic survey

We contacted (via email) HCPs working in NHS prostate cancer care (see Table 3) through their professional bodies (British Association of Urological Nurses, British Association of Urological Surgeons, British Uro-Oncology Group, Primary Care Urology Society) and commissioners via clinical leads and accountable officers of all Clinical Care Groups (CCG) in England and requested they complete an electronic survey of their current practice activity (S4 File). The questionnaire was developed and refined in partnership with a dedicated study patient and public involvement panel. Any missing or incomplete survey responses were supplemented by data from local provider webpages and follow-up letters, phone calls and emails. Letters were also sent to all CCGs within England. Links to our survey were also advertised to health care professionals on the social media site, Twitter.

**Table 3:** Survey respondent’s profession.

Profession	n
Allied Health Care Professional	3
Cancer Care Commissioner	3
Exercise Physiologist	3
General Care Commissioner	1
General Practitioner (GP)	7
Nurse	20
Oncologist	4
Physiotherapist	3
Urologist	35
Other	16
Total	95

### Analysis

**Fig 1:**
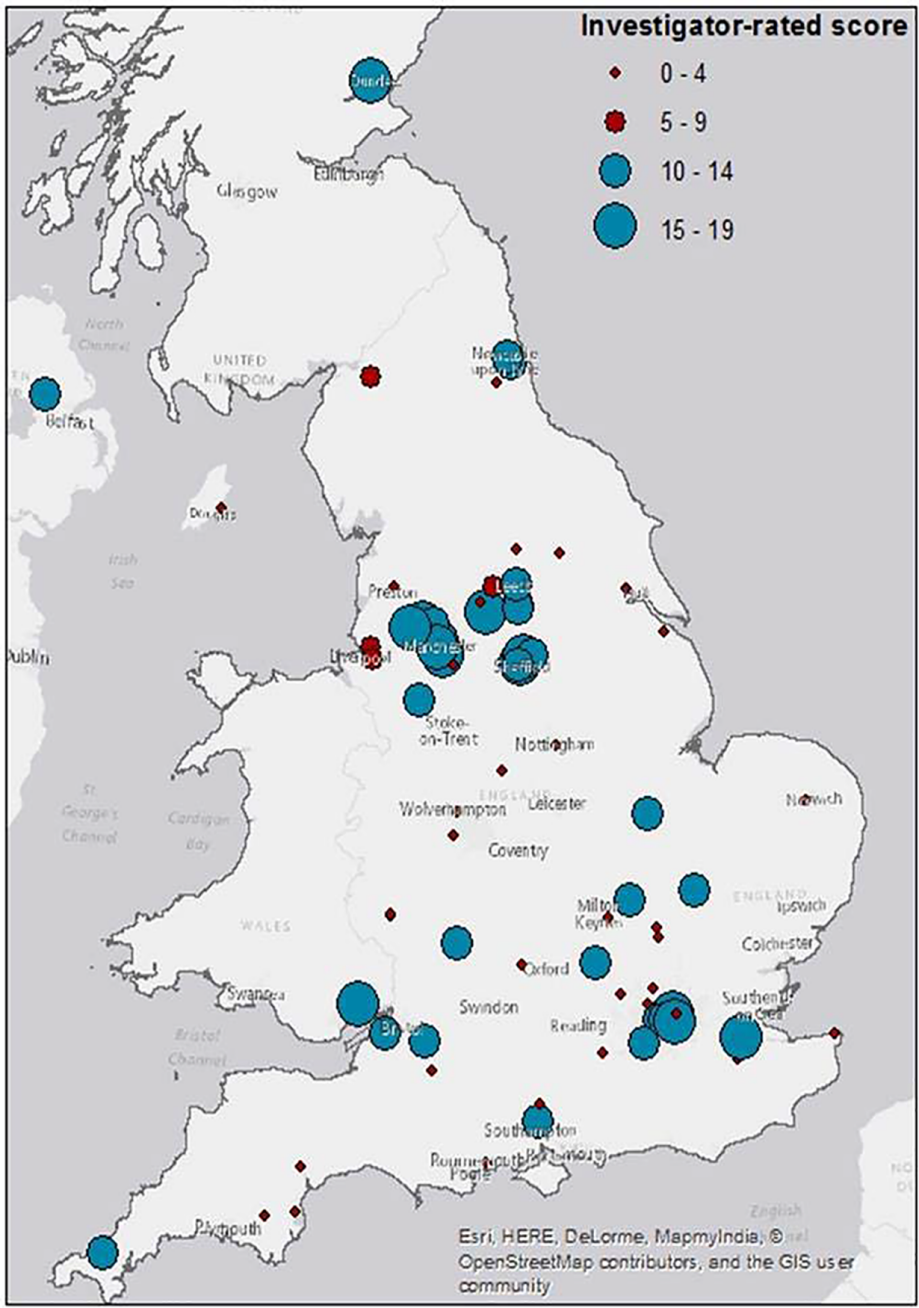
The distribution of survey respondents across the UK based on investigator-rated scores. **The blue (larger) circles indicate locations with good (15–19 score) or moderate (10–14 scor***e) capability to deliver exercise training according to NICE CG175*. *The (smaller) red circles indicate no (0–4 score) or limited (5–9 score) ability to deliver exercise training according to NICE CG175*.

Interviews and focus groups were recorded digitally and transcribed verbatim. Data were analysed using a thematic framework approach [25] with the aid of NVIVO software. Transcripts were double coded by study researchers. Key themes and sub-themes were identified. To determine variance in exercise training provision, two independent investigators rated the survey data by location in regards to likelihood of providing exercise services according to NICE guidance. Key indicators were: availability of local exercise referral schemes, multi-disciplinary team working, access to specialists in exercise prescription, supervised exercise provision, parallel behaviour change services and any specialist staff training programmes: please see Fig 1. The locations were mapped by postcode (ArcMap software v.10.2) using graduated symbols (circles) with a combination of size and colour indicating the grading.

## Results

### i. Semi-structured interviews with HCPs

Table 4 contains the full list of themes and sub themes arising from the 37 HCP interviews. For the purposes of brevity and clarity, key themes related to exercise provision with illustrative quotes are detailed below. All qualitative data used to construct themes and sub-themes can be found in S5 File.

**Table 4:** All interview themes and sub-themes.

Themes	Sub themes
Impact of ADT	Side Effects Quality of Life (QoL) and Coping
New NICE guidelines on exercise for men on ADT in the NHS	Awareness Standard of care
	Level of Conviction
	Clinical effectiveness
	Perceived benefits and purpose
	General Physical and Mental Health Management of Side-effects
Embedding in the NHS	Delivery of Programme
	Referral
	MDT Role
	Setting Feedback Evidence Base Championing Awareness of exercise programmes
Commissioning	Cost-Effectiveness
HCP Barriers	Referral process
	Resources Competencies Traditional Values
Potential solutions	Evidence Base
	Specialist further training
Patient Barriers	Impact of treatment Information Giving Worries and concerns Practicalities
Potential solutions	Necessity Support
Organisational barriers	Funding Resources
HCP Training	Practicalities Commitment Skills training Behaviour change and communication Referral Process Awareness

#### Impact of ADT: QoL

ADT was widely acknowledged as having significant adverse effects on social functioning and general QoL. A particular aspect that was identified was the impact on physical ability.

“… it's overwhelming and life-changing and devastating.” (CNS)

“…my patients … a lot of them were builders—they say that they don't do the heavy work anymore” (ONC)

#### Embedding in the NHS: Delivery of a supervised exercise training programme

Successful delivery of a prostate cancer specific exercise training programme as per the guidelines (NICE-CG175) was considered dependent upon having HCPs with the necessary skills. These include experience of exercise prescription, supervision and dealing with any comorbidities, to allow tailoring of the exercises to the individual’s needs and abilities. There was a general lack of consensus as to who could fulfil this role.

“… I think the actual assessing and doing the intervention should probably be, I would say, a physiotherapist probably because I am a physiotherapist.” (PHY)

“I'm not sure it's quite the physiotherapist's role …somewhere between a personal trainer and someone … at the leisure centre doing exercise classes and things.” (URO)

“I think someone with an interest … in this whole health benefit thing, and it could be a physio, could be a CNS.” (URO)

#### Embedding in the NHS: Role of the MDT

Integrating of exercise training within the MDT discussion of the care package was highlighted as a priority, without which successful implementation would be unlikely.

“Let's get serious on it (sic exercise training),… every place should be having a robust lead running MDT, ok. No place in this country should say that they don't have an effective MDT.” (ONC)

#### Commissioning: Cost-effectiveness

There was uncertainty as to whether exercise programmes for prostate cancer should receive funding from the NHS (cf ADT/chemotherapy). It was considered essential that these programmes should be embedded within the core service. Systemic benefits of exercise could make it cost-effective, given potential benefits in reducing psychological or cardiovascular complications of ADT.

“I think unfortunately the pragmatic side of it is unlikely that the NHS would be able to kind of fund all of it …” (CNS)

“I mean, if there's provision for a charity or if there's provision for any money by the NHS that's going to be … so if it's still free to the patient, I'd rather tap into it because you know, we're in austerity at the moment …” (URO)

“And it's not just going to help with fatigue, it's going to help psychologically. So perhaps referrals onto our clinical psychologist which probably costs a fortune cos they'll come down.”(CNS)

“…we won't have to perhaps see them so often because they'll feel better about themselves maybe; it'll reduce phone calls—we get a loads of patient phone calls, you know, just needing a bit of reassurance over the phone.” (CNS)

#### HCP barriers: Traditional values

It was considered that clinicians might be quite conservative as to the possible benefits of exercise training, particularly in elderly patients with metastatic cancer. These values were considered a potential barrier the integration of exercise training in standard treatment.

“I mean for some people, the idea to put 80-year old people on treadmills is close to torture …” (GP)

“You may get certain, you know, some doctors who just maybe don't see the value of it.” (CNS)

#### HCP Training: Behaviour change and communication

The importance of educating HCPs in behaviour change techniques, including specific approaches such as motivational interviewing, were highlighted as key to enabling integration of exercise training into the standard clinical pathway.

“I probably would need to do a little bit more work into behavioural change to look at really how you guide someone as expertly as possible to make positive health changes …” (PHY)

“So you have to have the ability to do that. I don't think any healthcare professional could do that without, certainly not without training at least.” (PHY)

### ii. Focus groups

The age range of participants was between 58–84 years. The majority were of white British ethnicity (96%), which was broadly reflective of the local population. The mean duration of ADT was 2.3 years (SD = 0.8). A number of the men suffered with comorbidities such as arthritis, hypertension, cardiac disease and diabetes. Godin Leisure-Time Exercise Questionnaire mean = 24.3 (SD = 21.4) and WHODAS II Questionnaire score mean = 14.4 (SD = 15.1).

Table 5 outlines the main themes arising from the 5 focus groups involving 26 men on ADT for prostate cancer. Illustrative quotes are provided for more detail below.

**Table 5:** All focus groups themes and sub-themes.

Themes	Sub themes
Experience of hormone therapy	Adverse effects of hormone therapy
	Impact upon QoL
	Impact upon identity
Coping	Approach coping style Avoidant coping style
Value of physical activity	Side-effects of hormone therapy
Adherence to exercise programmes	Barriers
	Solutions
Patient centred design of exercise schemes	Social contact Referral process Information giving Delivery Emotional support

#### Experience of hormone therapy: Adverse effects and impact on QoL

A wide range of adverse effects were reported. The most common included weight gain, hot flushes, fatigue, sexual dysfunction, the need to urinate more frequently and emotional lability.

“I hate it with an absolute passion. It’s because it’s changed my personality so much.”

“Hot flushes do make you tired. It shattered me completely.”

“I’m an emotional wreck. I’m not the person that my partner was marrying into.”

“Well, no sex for starters …”

#### Adherence: Barriers

Physical activity levels varied amongst individuals, but all the men stated that they would like to be more active. Co-morbidities, pain, fear of injury and lack of physical fitness were mentioned as barriers to exercise.

“But I’m wary now of falling over.”

“It’s not through lack of wanting to, … I’ve got half the haemoglobin floating around in my body.”

“I’m slightly out of sync, because at the moment I’m having chemotherapy and the chemo tends to have significant effects every three weeks. So I find there’s times where I just don’t do very much at all and the steroids kind of make me put weight on.”

#### Adherence: Solutions

Social interactions within group training sessions were seen as potentially important factors to help improve uptake and adherence rates of an exercise programme. Tailoring was seen as essential, particularly in men with other co-morbidities.

“In my opinion, it’s better in a group. I know some people like to do it individually, but from personal experience, it’s better in a group …”

“The key thing that would make me engage with it, is if it was individualised to me …”

#### Patient centred design: Referral process

The timing of referral was viewed as crucial. Views were mixed on when the programme should be offered to the men, however, it was agreed it should not be offered at the end of treatment. Offering a referral onto an exercise programme as soon as treatment is initiated was broadly supported.

“If exercise is proven to be beneficial to prostate cancer, then it’s beneficial as soon as you diagnose it.”

Whereas having a period of time to “digest” the diagnosis and information, then having access to the programme which would continue whilst they were receiving treatment felt more appropriate to some men:

“To answer your question, I’d rather on being diagnosed, find out what treatment they’re going to give me. Let me settle down a bit, let me come to terms with where I am and how I’m going to handle it, from both a personal and the family and wider circle of friends.”

The men felt that it was the consultant’s job to broach the subject of exercise initially, making them aware of the benefits of being active and making it specific to prostate cancer Having the appropriate written information on the benefits of exercise to support this and information on the programme itself would also be necessary. Further advice would be preferred to be given face to face by the nurse practitioner, with the nurse practitioner being responsible for going through any worries or concerns the men had. Being “sold” the exercise by the consultants, and the nurse practitioners would be best placed to provide encouragement:

“Yes, I agree with that. You have to be sold because you’ve been hit by the fact that you’ve got cancer and you need somebody to actually sell it and say “Look, we think this is going to work for you. I think you should try these exercises”.”

### iii. Electronic survey

A total of 95 responders from 79/154 NHS trusts provided data (51%). Anonymised survey response data and investigator ratings can be found in S6 File. From these 95 responses, 38 unique locations were rated as ‘moderately’ or ‘highly’ capable of delivering the NICE recommendation. Integrated descriptions of these sites based on survey data, interviews and follow-up phone calls can be found in S7 File. From follow-up phone calls to CCGs, HCPs and community exercise providers, a further 9 such locations were identified and rated, giving a total of 47 locations. NHS professionals and non-NHS sources provided 31 and 16 of these locations, respectively. Fig 1 depicts the distribution of these sites geographically. There is variability in duration, frequency and delivery with supervision of any kind being provided in 25 (53%) and twice-weekly supervised sessions in 8 (17%). Schemes lasting 12 weeks or more are reported by 24 (51%) and involvement of an ‘exercise specialist’ in the delivery in 21 (45%). There are only three prostate cancer-specific programmes and two of these described the exercise provision as being integrated into the usual prostate cancer care pathway.

## Discussion

Our data indicates there is minimal evidence that integration and provision of exercise training as per NICE CG175 is happening in practice. Whilst there is enthusiasm around exercise for men on ADT, HCPs involved in the prostate cancer care pathway find implementation of these guidelines problematic. The development of specialised education/training packages for HCPs for supporting exercise training and also the development of the evidence base were identified as factors requiring attention. Men on ADT asserted that their treatment does indeed have negative consequences on quality of life and that they would be enthusiastic about the provision of exercise training to address some of these issues. Any such service would need to be tailored to individual capabilities and existing co-morbidities.

It is important to acknowledge some key limitations of these analyses. The recruitment procedure did not allow us to calculate response rates for the survey as professional bodies acted as intermediaries in the identification process. We are not aware of any other recent studies undertaking similar analyses in the UK and as such these results provide unique data around the question of exercise provision for men on ADT according to the most recent iteration of the NICE guidelines. In addition, we relied on respondents to be a member of professional bodies or groups and to be actively reading emails from the group, which might have biased the sampling. We did not survey private sector providers, cancer related charities or voluntary agencies that might provide exercise referral schemes for people with cancer. Survey data from only 51% of NHS trusts could introduce the possibility of selection bias.

The provision of exercise therapy for long-term conditions is not a new concept. Exercise rehabilitation/training for cardiovascular disease is a useful comparison.[26] Meta analyses of randomised trials have reported benefits in terms of better overall mortality and cardiovascular mortality and reduced risk of hospital admission.[27,28] However, the delivery of exercise training with the fidelity that would reproduce the benefits reported in the clinical studies in NHS service is not a reality. Serious problems around staffing levels, multi-disciplinary involvement, exercise prescription (frequency, intensity and duration), parallel behaviour change support and the method of exercise delivery have been highlighted by senior academics leading the National Audit of Cardiac Rehabilitation.[29] Crucially, less than 5% of programmes in the UK state that they have a doctor as part of the multi-disciplinary team. With this fundamental lack of embedding of exercise services in core cardiac care, it is easy to see how they can become an afterthought and sub-optimally implemented (where done at all).

Men with prostate cancer consistently report unmet needs.[18] Previous analysis of prospective data taken from NHS services has highlighted that unmet needs in cancer survivors tend to stay unmet.[30] Despite the publication of this data (which is over a decade old), it is unclear where the innovation in cancer care pathways has taken place in the NHS—there are certainly very few multi-centre trials of supportive survivorship therapies that have provided both clinical and cost-effectiveness data. This is despite two key government policy documents in 2011 and 2015 highlighting the increasing importance of cancer survivorship.[31,32] Independent reports from CRUK assessing the implementation of the UK’s cancer strategies suggests that whilst cancer survivorship issues are broadly better appreciated in the NHS, dedicated services are seen as 'soft' and tend to be viewed as lower priority and particularly vulnerable in 'challenging financial climates'.[33] Indeed, it is often left to specialist cancer charities to fund enhanced cancer survivorship services including exercise referral services.

## Conclusion

There is substantial variability in exercise training delivery across the UK, with little provision available in line with the NICE guidance (CG175). Men on ADT for prostate cancer should be supported through treatment with dedicated, supervised exercise training and where available clinical teams should be making the appropriate referrals for their patients. Further research is likely needed to explore how to embed these services where referrals are not already happening.

## Supporting information

S1 File COREQ 32 item checklist. (DOCX)

S2 File Semi-structured interview schedule. (DOCX)

S3 File Focus group topic guide. (DOCX)

S4 File Online survey template. (DOCX)

S5 File Qualitative data. (DOCX)

S6 File Anonymised survey responses. (XLSX)

S7 File Site descriptions. (DOCX)
