# Supplementary material for: A multi-centre investigation of delivering national guidelines on exercise training for men with advanced prostate cancer undergoing androgen deprivation therapy in the UK NHS
Source: PLoS One. 2018 Jul 5;13(7):e0197606. doi: 10.1371/journal.pone.0197606 (PMC6033384; doi:10.1371/journal.pone.0197606)
Supplement: S1 File — (DOCX) [file pone.0197606.s001.docx]

**Table 1**

Consolidated criteria for reporting qualitative studies (COREQ): 32-item checklist

| **No** | **Item** | **Guide questions/description** | **Response** |
| --- | --- | --- | --- |
| **Domain 1: Research team and reflexivity** |  |  |  |
| Personal Characteristics |  |  |  |
| 1. | Interviewer/facilitator | Which author/s conducted the interview or focus group? | Rebecca Turner (RT)  Dr Eileen Sutton (ES)  Rosa Greasley (RG) |
| 2. | Credentials | What were the researcher's credentials? *E.g. PhD, MD* | RT - MSc BSc (Hons)  ES - PhD  RG - PhD student, MSc (Res), PGCert, BSc (Hons) |
| 3. | Occupation | What was their occupation at the time of the study? | RT - Research Assistant  ES - Senior Research Associate in Qualitative Methods & Patient & Public Involvement Lead  RG - PhD student |
| 4. | Gender | Was the researcher male or female? | RT - Female  ES - Female  RG - Female |
| 5. | Experience and training | What experience or training did the researcher have? | RT - Experience in group facilitation and qualitative research. Accessed a number of qualitative research courses ran by NatCen.  ES - Eileen Sutton is a qualitative researcher at the University of Bristol School of Social and Community Medicine with extensive experience of interview and focus group research with health professionals and men with prostate cancer.  RG - Accessed a number of qualitative courses ran by Sheffield Hallam University. Experience in conducting qualitative interviews as part of PhD study. |
| Relationship with participants |  |  |  |
| 6. | Relationship established | Was a relationship established prior to study commencement? | No |
| 7. | Participant knowledge of the interviewer | What did the participants know about the researcher? e*.g. personal goals, reasons for doing the research* | Occupations and reasons for doing the research. |
| 8. | Interviewer characteristics | What characteristics were reported about the interviewer/facilitator? e.g. *Bias, assumptions, reasons and interests in the research topic* | Researchers received external funding (NIHR) to undertake the work. |
| **Domain 2: study design** |  |  |  |
| Theoretical framework |  |  |  |
| 9. | Methodological orientation and Theory | What methodological orientation was stated to underpin the study? *e.g. grounded theory, discourse analysis, ethnography, phenomenology, content analysis* | Thematic framework |
| Participant selection |  |  |  |
| 10. | Sampling | How were participants selected? *e.g. purposive, convenience, consecutive, snowball* | Convenience sampling |
| 11. | Method of approach | How were participants approached? e*.g. face-to-face, telephone, mail, email* | Posters, letters, telephone, in Urology out-patient clinics and support groups. |
| 12. | Sample size | How many participants were in the study? | Interviews - 37  Focus groups - 26 |
| 13. | Non-participation | How many people refused to participate or dropped out? Reasons? | None |
| Setting |  |  |  |
| 14. | Setting of data collection | Where was the data collected? e*.g. home, clinic, workplace* | Workplace - Medical School, University of Sheffield or via telephone. |
| 15. | Presence of non-participants | Was anyone else present besides the participants and researchers? | Wives, partners or carers of the participants were welcomed to the focus groups. |
| 16. | Description of sample | What are the important characteristics of the sample? *e.g. demographic data, date* | Interviews  Demographic - Health care professionals within the cancer care pathway.  Focus Groups Demographic - men with advanced prostate cancer on hormone therapy. |
| Data collection |  |  |  |
| 17. | Interview guide | Were questions, prompts, guides provided by the authors? Was it pilot tested? | Yes, Semi-structured interview schedule and focus group schedule was produced by the authors. No it was not pilot tested, but it was approved by our patient and public involvement group. |
| 18. | Repeat interviews | Were repeat interviews carried out? If yes, how many? | No |
| 19. | Audio/visual recording | Did the research use audio or visual recording to collect the data? | Audio |
| 20. | Field notes | Were field notes made during and/or after the interview or focus group? | No |
| 21. | Duration | What was the duration of the interviews or focus group? | Interviews 30-45 minutes.  Focus groups 60 - 90 minutes. |
| 22. | Data saturation | Was data saturation discussed? | Yes, data saturation was discussed with the researchers. Once saturation was reached, no more interviews and focus groups were carried out. |
| 23. | Transcripts returned | Were transcripts returned to participants for comment and/or correction? | No |
| **Domain 3: analysis and findings** |  |  |  |
| Data analysis |  |  |  |
| 24. | Number of data coders | How many data coders coded the data? | 3 |
| 25. | Description of the coding tree | Did authors provide a description of the coding tree? | NA |
| 26. | Derivation of themes | Were themes identified in advance or derived from the data? | Derived from the data |
| 27. | Software | What software, if applicable, was used to manage the data? | N Vivo version 9 |
| 28. | Participant checking | Did participants provide feedback on the findings? | No |
| Reporting |  |  |  |
| 29. | Quotations presented | Were participant quotations presented to illustrate the themes / findings? Was each quotation identified? e*.g. participant number* | No |
| 30. | Data and findings consistent | Was there consistency between the data presented and the findings? | Yes |
| 31. | Clarity of major themes | Were major themes clearly presented in the findings? | Yes |
| 32. | Clarity of minor themes | Is there a description of diverse cases or discussion of minor themes? | No |
