# Supplementary material for: A multi-centre investigation of delivering national guidelines on exercise training for men with advanced prostate cancer undergoing androgen deprivation therapy in the UK NHS
Source: PLoS One. 2018 Jul 5;13(7):e0197606. doi: 10.1371/journal.pone.0197606 (PMC6033384; doi:10.1371/journal.pone.0197606)
Supplement: S2 File — (DOCX) [file pone.0197606.s002.docx]

| **S2 File**. **Semi-structured interview schedule**  **Introduction**  Thank you for your time in taking part in this interview. We are interested in your perspective regarding roles, responsibilities and training needs associated with providing supervised exercise programmes for men with prostate cancer on ADT. By supervised exercise, we mean a structured programme of exercise training delivered and overseen by a professional.  We would like to audio record the interviews but these will be completely confidential and all data will be anonymised in transcription and analysis. Can you please confirm you have read, understood and signed the informed consent form and are happy to proceed? |
| --- |
|  |
| **Questions** |
|  |
| - Can you tell me a little about your current role in the care pathway for men with prostate cancer on androgen deprivation therapy (ADT)? |
|  |
| - How do your patients cope with their cancer and ADT? |
| - What are the common adverse effects with this treatment, and which do you feel men find most bothersome? |
| - How would you say being on ADT for prostate cancer affects men’s quality of life? |
| - Are you aware of any non-pharmacological treatment or any supportive programmes designed to improve quality of life for men on ADT? |
|  |
| - What do you know about the role of exercise in treating men with PC? (knowledge) |
| - Are you aware of any guidance? Can you tell me what the NICE recommendations are as you see them? (knowledge) |
| - How do you feel about behaviour change strategies like looking at worries and concerns and setting goals? Do you think they have a role in exercise programmes? (beliefs about consequences) |
|  |
| **Current guidance recommends men with prostate cancer on ADT have access to supervised exercise which should include an exercise prescription as well as behavioural support such as goal setting and addressing worries and concerns.** |
|  |
| - How do you feel about this: |
| - As part of standard NHS practice? |
| - Should this be separate from NHS care? |
|  |
| - What is your organisation already doing with regards to exercise for men with prostate cancer? (memory, attention, decision) |
| - Are you currently involved in setting goals with your patients and do you follow up on whether these are achieved or not? |
| - Are you aware of any exercise programmes for other patient groups? How beneficial do you think exercise/exercise programmes would be for your patients with prostate cancer? |
| - Who’s role would you see it as to i) make referrals for exercise programmes ii) delivery of exercise? (social/professional role identity) |
| - Should this take place in primary/secondary care/community/outpatient settings (who specifically?)  **Probe* who should introduce idea to patient, delivering exercise (prescription & behaviour change elements), following up with patients the amount of exercise being done. If you do not see it as your role, can you elaborate as to why and who might be better placed?* |
|  |
| - Have you been involved in referral or delivery of exercise programmes to any other patient groups in the past? |
|  |
| **Our research team are hoping to evaluate how a 12 week, or 12 month, supervised exercise programme could be delivered in the NHS for men on ADT. This will require professionals in your role to support this process. That might involve making referrals, delivering the exercise programme and providing specialised behaviour change support. How would you feel if one or more of these elements became part of your role? (social/professional role identity, emotion)** |
|  |
| - What applicable skills do you think you currently have? Do you think you’d be able to new relevant learn skills (what training would that need) (skills) |
| - Given training do you think you’d feel confident in doing this? (optimism) |
| - How difficult or easy do you think it would be for you to do? (beliefs about capabilities) |
| - Would it be something you’d like to do (all/part/none)? (goals) |
| - Would it be compatible with how you see your role? (social/professional role identity) |
| - How do you think your colleagues/seniors e.g. consultants/managers would respond, would this help or be a problem? (social influences) |
| - Would there be capacity to support a 12 week or 12 month programme? What would help to facilitate capacity? (environmental context and resources) |
|  |
| - Given people were trained and happy to do this what barriers might there be to putting it in place from your point for view? Does this differ between a 12 week and 12 month commitment?(environmental context and resources) |
| - Practical/resource |
| - From staff, patients, systems? |
| - What do you think would happen if you did take this on? (for self/patients) |
| - If this did become part of your role is there anything which would make it more likely you’d do it (incentives), what would these look like? (Reinforcement) |
| - Would there be systems that could help monitor if it’s being done, make it easier? (Behavioural regulation) |
|  |
| If the exercise programme was put in place what do you think patients would think about it? |
| - Positive/negative reactions |
| - Barriers to attending, |
| - What would make them more likely to attend and maintain their involvement? |
|  |
| - Going back to training, if this was to take place, would you be able to undertake training sessions in your current role? |
|  |
| - What would be the best way to deliver the training? |
|  |
| **Probe* format, length, time of day, location, number of sessions, duration of sessions, practical element with supervision, including videoing?* |
|  |
| - Would you be prepared to do things like homework, keep a reflective journal? |
|  |
| - If we were to develop a training programme with a view to implementing an intervention would you be interested in taking part? (Intention) |
| - If not, why not? |
