# Supplementary material for: A multi-centre investigation of delivering national guidelines on exercise training for men with advanced prostate cancer undergoing androgen deprivation therapy in the UK NHS
Source: PLoS One. 2018 Jul 5;13(7):e0197606. doi: 10.1371/journal.pone.0197606 (PMC6033384; doi:10.1371/journal.pone.0197606)
Supplement: S3 File — (DOCX) [file pone.0197606.s003.docx]

| **S3 File. Focus group topic guide**  **Introduction**  We would like to audio record the focus group but these will be completely confidential and all data will be anonymised in transcription and analysis. Can you please confirm you have read, understood and signed the informed consent form and are happy to proceed?  What does Physical activity mean to you?  **Develop collective definition** |
| --- |
|  |
| - How many of you would say you are physically active now? |
|  |
| **Probe* use community facilities, exercise referral schemes, commercial gyms, home, other?*   - Generally, how does your prostate cancer affect you and your quality of life? - Thinking about hormone treatment, how does this affect your quality of life, what are the most bothersome effects? |
| - Have you ever heard of a role for physical activity in men with prostate cancer? Tell me what you’ve heard/experienced.   Where was this from?   - Were you give any advice or information about physical activity by your health care team? If yes probe – who from GP, nurse, urologist / what advice were you given / when were you given this advice? - If you weren’t given advice on physical activity would you like to have been? Who do you think should give advice / when? |
| **Recent guidelines have suggested being more physically active can be useful for men with prostate cancer** |
|  |
| - What benefits do you think you would see from physical activity for your prostate cancer?   **Probe* Research has shown us that men with prostate cancer on ADT suffer with side effects such as fatigue, reduction in sexual activity and functioning. Do you think physical activity would improve this?* |
|  |
| - How important do you see physical activity for your health/ recovery from prostate cancer? |
|  |
| - Would you have any worries or concerns about being more physically active? |
|  |
| - If there was a programme available through the NHS, how many times a week do you think you could undertake some supervised physical activity? For how long could you do this? e.g. up to 12 months? |
|  |
| - What would be the best format? |
| - Group, individual, with partners? |
| - In hospital, in the community? |
| - Some formal sessions, some at home? |
| - With your partner? |
|  |
| - Who would you like to manage and support your physical activity programme? |
|  |
| - Who would you like to discuss the idea of physical activity first, including benefits, possible problems worries and concerns? |
|  |
| - How best would this be – face to face, over phone, email? |
|  |
| - If a programme was set up:   - Why would you attend?  - What would make you attend/not-attend this programme?  - What might get in your way of attending |
|  |
| - What sort of information would you like to know about the programme prior to the start of the programme? (discuss expectations, benefits, intensity of exercise, access, duration etc) |
|  |
| - How soon would you like to start the programme once invited/referred? |
|  |
| - What would make it easier to keep coming? |
| - Is there anything else you would like to add or discuss? |
