# Supplementary material for: A multi-centre investigation of delivering national guidelines on exercise training for men with advanced prostate cancer undergoing androgen deprivation therapy in the UK NHS
Source: PLoS One. 2018 Jul 5;13(7):e0197606. doi: 10.1371/journal.pone.0197606 (PMC6033384; doi:10.1371/journal.pone.0197606)
Supplement: S4 File — (DOCX) [file pone.0197606.s004.docx]

**S4 File.** **Online survey template**
