# Supplementary material for: A multi-centre investigation of delivering national guidelines on exercise training for men with advanced prostate cancer undergoing androgen deprivation therapy in the UK NHS
Source: PLoS One. 2018 Jul 5;13(7):e0197606. doi: 10.1371/journal.pone.0197606 (PMC6033384; doi:10.1371/journal.pone.0197606)
Supplement: S5 File — (DOCX) [file pone.0197606.s005.docx]

**S5. File**

**All semi-structured interview themes and sub-themes.**

**Impact of ADT**

The treatment was acknowledged as having a number of adverse effects by the health professionals, which were suggested to impact upon a man’s quality of life, health and social relationships.

- **Side Effects**

The impact of physical and psychological side-effects from ADT were acknowledged by all the health professionals, these included hot flushes, fatigue, weight gain, depression, changes in confidence, erectile dysfunction and a loss of muscle mass.

*“The erectile dysfunction that it causes irrespective of whether or not they were sexually active, the loss of erectile dysfunction has an impact on a man's psychology right.”* (ONC)

*“These things go off a bit of a cliff when they start the hormone therapy, so they've got a sense of what they're normally like, um, and they very quickly get a sense that they're different on hormones.”* (GP)

*“I do get patients even now who've been established on hormones for a long time who are only just starting to realise that it has a long-term effect on things like bone health and cardiac health.”*  (CNS)

*“Fatigue, hot flushes, hot flushes are probably the top one ... um .... a change in mood, so kind of more low mood. Um ..... I often see men for urinary urgency and frequency.”*  (PHY)

- **Quality of Life and Coping**

The impact that ADT has upon men was well recognised, particularly effects on quality of life, ability to work, social lives and interpersonal relationships. Men not being able to continue to work anymore due to treatment, was suggested to be an issue, but an issue that is rarely discussed during clinic.

*“it's my patients who have told me, particularly a lot of them were builders - they say that they don't do the heavy work anymore, like more they supervise it and their sons do it or some partner does it, you know”* (ONC)

*“I don't think perhaps health care professionals really realise the impact ADT has on people's ability to work effectively. So it's unawareness really and unawareness of the importance of quality of life, not being to work is a big issue.”* (PCP)

A knock in confidence and low self-esteem was suggested to have an impact upon the men psychologically, causing emotional distress.

*“The really biggest one is just the fact that patients' confidence is knocked, you know, whether that's confidence to exercise or confidence to socialise or, you know, do things with family”* (PHY)

*“Because it's so sad that they can be in such an emotional and physical pickle really with their side-effects of their treatment”* (PHY)

*“Some, you know, it's overwhelming and life-changing and devastating.”* (CNS)

It was recognised that men seemed to differ in how they coped with the diagnosis and the treatment, with some tolerating the treatment well and others really struggling. It was suggested that some men display denial coping strategies and are less active in seeking help or discussing issues, particularly with their doctor or consultant, compared to their nurse. It was also expressed that this may be due to males tending to be less proactive at seeking medical help and talking about their health compared to women.

*“I think actually men particularly with prostate cancer are less good at seeking help, they don't complain about ... particularly they don't complain about sexual problems or emotional problems because it's not the way they're wired up, is it, they just don't.”* (GP)

*“Very different, some of them cope well, others don't. I think that's very much dependent on the context.”* (GP)

*“I think it varies, I think for most people it does affect their quality of life detrimentally; but I still think that what they do is they downplay, they kind of just say well I 'm used to it now, I just get on with it, I haven't got any other choice or they don't really appreciate that there are actually things that we can do.”* (CNS)

**NICE Guidelines CG175 1.4.19**

Offer men who are starting or having androgen deprivation therapy supervised resistance and aerobic exercise at least twice a week for 12 weeks to reduce fatigue and improve quality of life. **[New 2014]**

- **Awareness**

The majority of health professionals interviewed were aware of the recent NICE guidelines and appreciate that the guidelines are based on the best available evidence. Awareness was normally due to specific, personal interests within this area, with most health professionals believing that the guidelines were not being carried out nationally.

*“I think ... my knowledge of NICE guidelines .... It’s normally very thorough and it will based on best available evidence ...”* (URO)

*“I'm aware of them; I'm not convinced that that's happening … I am, but probably only because of my interest, if you see what I mean.”* (GP)

- **Standard Care**

Despite awareness of the NICE guidelines and perceived benefits, the general consensus was that the NICE guidelines were not being delivered. Some clinicians offered general diet and exercise information at consultations, but this was subject to individuality.

- **Level of Conviction**

It was expressed that there was a slight doubt about how robust the evidence is for these NICE guidelines compared to other NICE guidelines, due to reasons such as it being difficult to document robust outcomes from exercise. It was also expressed, that the NICE guidelines are *“only guidelines”,* so issues may arise when trying to convince the funders they are responsible for paying for this. Therefore, even due to the NICE guidelines having the role of setting the ‘gold standard’ for health and social care within the UK, it was stated that there were still doubts about the strength of them, which could impact upon following such guidance.

*“One can be sceptical and say that the evidence that they looked at for exercise and put in the guidelines is not as robust as some of the other evidence that is out there and they're forgot to mention those things in the guidelines right?”* (ONC)

*“Very difficult to document what difference exercise made to the outcome of a patient, ok.”* (ONC)

*“Whether I can persuade my organisation when the time comes when they have to pay for it, um .. That will be an interesting time to see."* (CCG)

- **Clinical Effectiveness**

Health professionals identified the NICE guidelines as being important, possibly even essential and extremely beneficial to the men. Particularly due to people living longer with cancer, it was expressed more resources should be put into this patient group.

*“I think it's good for the gents who are suffering, because they realise if they're to exercise that it is ok and, you know, a lot of people hear that C word and it just scares them to death and they just want to go and hide away.”* (ES)

*“Well, it ought to be because, you know, the idea is that cancer is hopefully not something hopefully that you die of, at least you don't die of it straight away. Because there's an awful lot of, you know, people are living longer with cancer so there should be more funding to adequately look after those patients really; at the moment I think that's quite scanty.”* (GP)

Whereas some health professionals believed there were other interventions, programmes and drugs etc. that also need funding, so it’s hard to prioritise what is more important and felt uncomfortable to answer this.

*“that would do injustice to other things and it's the usual argument - what's most important: is it exercise for people with mental health problems, is it the care for this group of patients, is it care for young women with eating disorders and that's always balanced against each other and I can't say what's more important and where should be healthcare allocation and funding, I can't ... find it a very difficult conversation.”* (GP)

- **Perceived Benefit and Purpose**

It was agreed that an exercise programme as such had a place within health care, as there was perceived benefit and purpose of the programme.

*“I personally think it's a fundamental aspect of healthcare so, you know, I think it would be hugely beneficial if we had more access to it.”* (GP)

*“if it was a drug, exercise would be being prescribed all the time ...”* (GP)

- - **General Physical and Mental Health**

Supervised exercise was viewed as having many benefits for the men, physically and psychologically. It was expressed, the benefits of exercise are well-known, specifically for cancer patients, but for people in general too. Exercise was suggested to help improve cardiovascular health, reduce BMI and decrease mortality, as well as help to manage the side-effects. It was viewed as a way to help to improve the men’s quality of life, their social lives, and help them to potentially get back to work, if necessary.

*“Initially I think it's more normality, I think that's the first thing they see that they're actually getting back to some kind of normal lifestyle and I think that's the biggest one for them.”* (ES)

*“Um ... well I think there's increasing evidence that exercise decreases death rate, not just prostate cancer but cardiovascular fitness and cancer, you know there is a link. And also, perhaps even more importantly, body mass index. So your chances of survival and good quality of life increase massively if you've got a normal body mass index and you've got cardiovascular fitness, so yes.”* (GP)

*“I think an increased feeling of well-being, an increased quality of life, reduction in cardiovascular morbidity and mortality.”* (URO)

- - **Management of Side-effects**

Some of the health professionals saw exercise as a way to manage the side-effects of ADT, with the consultants deeming themselves responsible for helping to reduce the side-effects of the treatment they have initiated. Having exercise as part of their cancer care pathway, will help ensure they are finding a way to manage their side-effects correctly.

*"I think if you as a doctor are starting a patient on treatment side-effects … it's onerous on you to try and do your best to reduce the risk of the side-effects and do something about them if you can."* (URO)

*“to sort of masculate them a little bit more by sort of encouraging them with exercise is ... and seeing the feedback that they give at the end is great really, and it's giving them control because, you know, it's quite a man-thing isn't it, sort of needing to be in control a little bit more.”* (PHY)

**Proposed Exercise Programme**

- **Roles**
  - **Delivery of Programme**

HCPs with existing skills around exercise prescription and delivery were felt to be important, given the need to tailor the exercises to the individual’s needs and abilities, but also be aware of any other comorbidities the patient may have, although exactly which HCP that might be, was not clear.

*“With people who are specifically trained in, you know, teaching exercise but specifically for people who, you know, some of them are quite elderly, you know.”* (CNS)

*“I think someone with an interest ... in this whole health benefit thing, and it could be a physio, could be a CNS.”* (URO)

*“They have to understand how to prescribe exercise and modify it and I think a lot of other roles would find that difficult to do safely and well.”* (PHY)

There were mixed views about whether a health professional should be running the exercise programme itself, but it was agreed that it would require someone who was qualified to run exercise for cancer patients. A physiotherapist was suggested by some health professionals, as they had knowledge of anatomy and physiology.

*“I'm not sure it's quite the physiotherapist's role but it isn't really whether they'd be able to help with that, I mean, you know, somewhere between a personal trainer and someone doing some, you know, at the leisure centre doing exercise classes and things.”* (URO)

The complexity of the patients however, suggested that a physiotherapist may need to be involved in the tailoring of the programme and doing assessments, whereas an assistant could supervise the exercise programme. So utilising staff to the staff to be the best possible way, was suggested.

*“I mean the actual running of the exercise I think could be ... we have like rehab assistants, but I think the actual assessing and doing the intervention should probably be, I would say, a physiotherapist probably because I am a physiotherapist.”* (PHY)

*“But utilising those patients that aren't as complex and utilising the personal trainers that have done the CAN rehab which is the Level 4, so that would be a great next step into it. But I'd definitely say there are complex people and that's why physiotherapy is really useful.”* (PHY)

*“I think if someone is going to be delivering exercise groups, they have to have been trained in that patient group, the problems they have ... they have to understand ... and this is why the physios are good at delivering it ... they have to understand anatomy, they have to understand physiology.”*

- - **Referral**

A number of the consultants saw the referral process as part of their job role, however others thought this task would be best placed with the Clinical Nurse Specialists due to their relationship with the patient and having more dedicated time with the patient, so could therefore discuss any concerns the men may have. However it was agreed that it would be most beneficial and in the patient’s best interest, if everybody in that patients care, could be involved in the referral process or discussion. Having communication and awareness amongst the team of the proposed exercise programme was suggested to be the most effective, with the responsibility not just sitting with one individual.

*“... I think everyone involved in that patient can say, have you signed up to the programme? And it should be the whole team doing it really. Because, in a busy surgery, the GP will forget and, giving the prostate injection, the nurse will forget and the consultant in hospitals is the least likely to remember. So I think it needs to be everybody saying, just like when you develop diabetes you get referred to the dietician, I think it should just be ... everybody knows about it, everybody says have you signed up?”* (GP)

*“I would have thought it would be good coming from the clinical nurse specialists, I mean they have a lot of contact with patient through this group of men locally.”* (URO)

*“No I think the nurse specialist. Because I think the consultants' time is always so much more limited and I think the reality is that in a lot of cases they're really there for kind of the mental side of things, and as CNSs we tend to know the patients better and I certainly think, you know.”* (CNS)

Having the consultants promoting the exercise programme and them explaining the benefits of the programme was expressed to be important and could help to improve adherence.

*“what you need with men I think is that you need the strong advice and direction from the oncologist or the consultant to say this is where you're going, this is your treatment.”* (PHY)

- - **MDT Role**

Being part of an MDT was expressed as being crucial for good patient care. Working together as a team, with everybody having the same understanding was suggested as being necessary to help empower patients. The MDT was highlighted as being a key dynamic within this exercise programme, the MDT was suggested to be able to help identify patients for the programme and help to raise awareness. Having all the Urology team all on board was highlighted as a priority in order to raise awareness and develop the programme successfully.

*“We work as a team, we talk about treatment as a team, we empower patients as a team.”* (ONC)

*“Let's get serious on it, so what you need to do is, you need every place should be having a robust lead running MDT, ok. No place in this country should say that they don't have an effective MDT.”* (ONC)

*“We have a team meeting every 6 weeks, so I suppose it would just be mentioning that at team meetings, you know, just reminding everybody are they still making referrals into the training programme, I suppose that would be a way of ... that's the only time we're all together really, every 6 weeks.”* (CNS)

*"Yeah, and we've tried to link with oncologists, any other appropriate, sort of, MDT leads .... I think .... yeah, what's tended to happen is, you know, our advisor has gone round to remind people that they're there and that the project's there, and then they get a little influx of referrals and then it all goes quiet again."* (CCG)

- - **Follow up**

Then being able to follow up the patients, back in clinics and at appointments by the health care professionals was suggested to be an idea, some of the health professionals were happy to do. This would involve, discussing feedback from the exercise programme that will have been reported and documented by the professionals running the exercise programme such as blood pressure reduction. Whereas others were not and saw the referral process was where their job role ended in regards to this programme.

*“Yeah, it would be quite nice to talk about it, because a lot of the time you're just seeing them with a blood test and so you've got to string out and you look at the blood test within 2 seconds, you know, which way is it going, so it's nice to have something to talk about.”* (URO)

*“Yeah, we're used to looking at numbers and trends over time ... doctors generally are programmed that way so we like looking at numbers over time, and if you give us that information, it's the way we see information all the time. So we'll engage much more with it, we'll be able to say you know, we'll be able to have a dialogue about it.”* (URO)

*"I mean, I guess my role would be to say as I see my role in discussing treatment with the patient and combatting side-effects is to advise them that we know that exercise helps to combat fatigue, you know, this is available, these are the classes and, um, I don't see my role would be more than that really."* (URO)

- **Referral Process**

It was expressed that the timing of the referral and the referral process were really important parts of the pathway to consider.

It was suggested that a referral onto an exercise programme for this patient group should be initiated at the start of treatment to get the momentum going, help with motivation and adherence. It was expressed that it was common for patients to state they wish they had access to supportive, non-pharma logical programmes at the start of treatment.

*“The feedback we had from patients was always, you know, they wish they'd been given advice about exercise and sort of diet and how to access counselling if they need it ... they wish they'd been given it sort of early in their cancer journey rather than at the end.”* (PHY)

*“I think they should start straight away to be honest with you or they lose the impetus.”* (URO)

*“Really I think if we're to get the benefits from it, it should be encouraged from the moment someone commences hormone therapy ...”* (GP)

However, others suggested that an initial referral may overwhelm the men, due to having just being diagnosed with prostate cancer. Therefore giving the men time to adjust to their diagnosis and start their treatment was suggested. Also, it was expressed that at 3 months the men would normally be starting to suffer with side effects of ADT, so may be keener to start an exercise programme, so therefore waiting 3-6 months before a referral was desirable.

*“So usually when I see them back here for their injection, they've had time to think about things, they've had time to review the information they've got, we're going through what happens next in terms of their follow-up and their treatment and they're a bit more amenable to kind of right, you know, what's going to happen?”* (CNS)

*“it's usually about the third or fourth treatment they get when they start to get the severe joint pain, the weight gain can be quite steady but there's definitely a drop after about 3 treatments to the joint pain that they get, and when they start to develop the hot flushes as well that's when there can be a drop in activity levels cos that'll cause a hot flush and they really don't like the feeling of the hot flushes.”* (ES)

It was suggested that an exercise referral should be promoted as part of the patient’s treatment within secondary care. For example, the referral for the exercise programme would be initiated at the same time as the ADT prescription. It was recommended that the exercise should be endorsed and promoted by the consultant as part of their treatment.

*“For every single patient in a timely manner then whichever clinician is seeing the patient really, so either the nurse or, you know, either the doctor or CNS that's seeing them.”* (CNS)

*“Yes, well I think it should be a compulsory part of the treatment. So at diagnosis, it's handed out as a we're going to operate on you or we're going to give you hormone injections, we're going to monitor you every 6 months and you're going to attend this course.”* (CNS)

*“What I'd like to imagine is that when you write a pharmacy prescription for an ADT, the referral comes off the back of that. So you almost change the prescription to say ADT with exercise.”* (URO)

- - **Concerns with referrals**

Concerns with referrals were identified, ensuring the programme was well ran and organised was believed to be important as there was concern that if there were any problems with the programme, the onus would come back to the doctor.

*“The worse thing would be to have the patient pitch back in 3 months with the PSA test saying I'm otherwise doing well but you talked about exercise, nothing happened, I phoned them, they said no no, this is not for you, this is ... you know, and then the whole chaos starts and then they lose confidence in the system.”* (ONC)

Having a referral process that was easy and not time-consuming was preferable, and this would probably increase referral rates. Offering alternative methods such as phone call or email would also ensure the health care professional was able to uptake a method that was easiest for them.

*“If the referral route was easy and not time-consuming, yeah, yeah. Again, it comes down to time; the hospital staff are just so busy, so it would just be ... and that's what we try to do here ... is I give health professionals a variety of different methods to refer to me so it could be by email or by a telephone call or paper referral, whichever is easiest for them.”* (PHY)

*“You know, we're faced with so many myriads of forms and inclusion ... you know, you can only come if you meet these criteria ... it needs to be a simple process just to avoid putting people off by the actual process being too complicated and time-consuming.”* (GP)

- **Delivery**

Running exercise programmes within a group setting was suggested to be beneficial to the men, so they can gain support from one another. It was, however debated whether the exercise programme would be best ran alongside other cancer groups or health conditions or whether it would be best delivered in a group specific to prostate cancer.

*“Isolation kills as well .... and that's why, if you're on hormones and you're isolated, nothing can be better than buddying up and getting together a programme like this ....”* (URO)

*“it's like a support group without the name, because they all help each other out with the treatments, the all talk to each other about how their partners dealt with it and stuff, so that's essential in that type of thing because it's getting them to do an exercise base which is quite manly and they accept it.”* (ES)

*“I can see the point in having, you know, a cancer group. It's not a great word obviously, but when they're going through chemo and hormone replacement therapy and stuff like that, they're all aware of that. But I think equally to integrate them into other groups as well, it's fine. I think it depends on the individual.”* (ES)

However, it was suggested that it needs to be considered that some men may not enjoy a group setting and this was something that needed to be noted.

*"And I guess there's two sides of the coin: sometimes people like to get together with a peer group; other people don't want to, they don't want to be identified as a cancer patient and you go in the corner over there with the other cancer patients"* (GP)

A highly supported programme was suggested to be the most beneficial to the men, as they can be guided through the benefits of exercise and gain confidence. Also, a well-supported programme was suggested to have better adherence rates. Having behaviour change support integrated within the exercise programme was stated as being very important and something that some of the health professionals were very familiar with. Behaviour change support such as goal-setting, discussing concerns and taking objective measures with the patient was considered to be important to achieve progress and overcome barriers.

*“it's about progressing those on so they're moving forward or if there's still problems coming up or we can identify that and try and find ways to work around it.”* (PHY)

*“Because it's so supported that they gain confidence and they understand the exercise; like, we're very big on explaining why they're doing exercises so we don't just say right, get on a treadmill and I'll see you later!”* (ES)

It was expressed that it was necessary to consider that the exercise programme may have to be ran in different models, in different parts of the country, so more individual exercise programmes may be necessary rather than group programmes in rural communities, as there may not be enough people to fill a group.

*“Yeah, that's what would work here, you know, I mean I'm sure it would be a bit different in a big city but ..um ... I don't know if you can picture Shropshire? It's a huge rural county with not many towns in it - it's probably got more sheep than people”* (CCG)

- **Setting**

Having an exercise programme ran within the community was preferable amongst the health professionals due to facilities differing trust by trust, so being able to use local community facilities that are already available would be ideal. Other reasons why having the intervention ran in the community was preferable was so patients could easily access the facilities, patients didn’t have to travel far to attend and parking would not be an issue as it is at hospitals.

*“I mean, we have patients come from all over, but they ... some travel a distance and, you know, they're much more likely to adhere if they have something much more local, you know, down the road or within 10 or 15 minutes ...”* (URO)

*“I think in the community, the other aspect is not just the logistics of it and the ease of it, but also I think that it promotes the more sort of … and you're likely to be working with a similar population”* (ONC)

*“When it's done in the community it's away from hospital, so it's more relaxed isn't it, because a lot of people get quite wound up about coming into hospital. So I suppose if it was in the community it would be more of a sort of ... yeah, it probably would be better in a way because it would be away from the hospital and also we have such terrible parking issues here and that winds everybody up!”* (CNS)

It was also expressed that this could be viewed as slightly separate from medical care and decrease stress associated with attending the hospital, if it was ran in the community. A community ran initiative would also encourage independence and confidence, with less reliance upon the medical staff at the hospital.

*“I see this slightly separate from that and I think it would be refreshing for patients to see that as a different sort of aspect and perhaps have less medical input and more, you know, this is your exercises and that's that.”* (URO)

*“I mean I think it's a good thing to try and work towards getting these things out in the community, because we want them to be able to embrace taking that forward and having the independence and confidence to do that in the community … we don't want them to be feeling so reliant on the hospital.”* (PHY)

*“I don't think people want to associate coming back to the hospital with getting on with life. People get stressed coming to hospital, we don't want that.”* (URO)

Funding was suggested to be a potential issue to where the intervention is placed, with this being the main issue to consider. Something that is local and easily accessible was preferred though.

*“It’s often a funding question and not a geographical question. In London, our hospital is in the middle of the borough, it's accessible, and it’s closer to home. Yes, it's in a very expensive environment, but if you are in the peak district or north Wales of course it's different.”* (GP)

*“It probably depends who's got the money, who has got the funding”* (GP)

- **Feedback**

Receiving feedback from the exercise programme, in terms of objective measures such as BMI, blood pressure, exercise levels and improvement of quality of life etc., was suggested to be important for the doctors and for the patients care. The idea is that it will allow the health professionals to be able to engage with the patients regarding the feedback. It was suggested that the patient’s records were kept up to date and information was provided on their progress.

*“I should be able to have a very simple summary, like, not a 6-page letter that on week 1 they did this or ... but just a summary saying week 1 to week 12 exercise, you know, some like plotted graph saying from 10 minutes he went up to 30 minutes' graded exercise … So I know then that that can feed into me being more positive about the patient maintaining that.”*  (ONC)

*“Sort of asking them, you know, how much more energy they feel they've got, you know, do they feel ... what their side-effects are like, but I guess if you do it straight away from starting the hormones, it would be difficult then to know how you've combatted the side-effects, if you like, cos they won't have had time to happen.”* (CNS)

*“Yeah, we're used to looking at numbers and trends over time ... doctors generally are programmed that way so we like looking at numbers over time, and if you give us that information, it's the way we see information all the time. So we'll engage much more with it, we'll be able to say you know, we'll be able to have a dialogue about it.”* (URO)

*“Yeah, it would be quite nice to talk about it, because a lot of the time you're just seeing them with a blood test and so you've got to string out and you look at the blood test within 2 seconds, you know, which way is it going, so it's nice to have something to talk about.”* (URO)

Ensuring that the exercise programme is fully embedded into the cancer care pathway was suggested to be crucial, for adherence and referral rate, to also ensure the patients were given access to the programme.

• **Evidence Base**

Having a good evidence base was identified as being important to embed and add support to such a service. Having good links with the MDT and out patients department was expressed as being necessary to help to embed a service successfully.

“I think always it's really nice to have an evidence base which we've definitely got and it's growing and then it would really key to have the consultants and CNSs keen to engage.” (PHY)

“so it's just getting the awareness of all the different groups that are out there now and just keeping people updated on how easy it is to refer.” (PHY)

• **Championing**

Having the programme championed by the consultant and led by them was suggested to be the most effective in embedding such a service. MDT leads were identified as being the key individuals who would help to implement such a programme and help to champion the project. Approaching MDT leads about an exercise intervention for men on ADT was suggested the best way to help integrate the programme and gaining support.

"Yeah, and we've tried to link with oncologists, any other appropriate, sort of, MDT leads .... I think .... yeah, what's tended to happen is, you know, our advisor has gone round to remind people that they're there and that the project's there, and then they get a little influx of referrals and then it all goes quiet again." (CCG)

“GPs are not going to be prescribing this off their own bat, so I think it needs to be consultant-led supported by everybody else; I don't think it should rely on posters in the waiting room, I think it needs to be the consultant says this is the form, please fill in the details and give it to me and you are in the programme. I think all consultant urologists are the key.” (GP)

"Yeah, um ... I think we've lacked a really strong champion within our cancer centre for this, so they have a role, they have like a lead cancer clinician role and a lead cancer nurse" (CCG)

• **Awareness of exercise programmes**

Having all health professionals who are involved in the patients cancer care to have the same understanding and awareness of the proposed programme. Raising awareness to all these different groups of health professionals was expressed to ensure the programme was embedded and referrals were made from numerous individuals.

“Absolutely, to speak to different groups, that's the key I think. Nurses, a lot of the injections are done by the practice nurses, I would address them. Um, psychologists, everybody who is working in this field of cancer and older people, and geriatrics, intermediate services, old clinics.” (GP)

“I just think raising awareness as well, just so everybody knows it's available and it is something.” (CNS)

**Commissioning**

HCPs felt that such programmes would be ideally, best funded by the NHS: the likelihood of this in the current economic climate was contentious however. Alternatives such as charity funded schemes were suggested, but would require better embedding in the current service.

*“I think ideally it should be NHS because it's health promotion, it's part of their treatment, I think their treatment now shouldn't just encompass what we're doing medically, it should encompass everything else that goes with that, so general health and well-being. I think unfortunately the pragmatic side of it is unlikely that the NHS would be able to kind of fund all of it, that's where the difficulty often is.”* (CNS)

Having a charity ran initiative, which was extremely well-supported by the NHS and embedded in the cancer care pathway, was suggested. Having the programme ran by a charity, in conjunction with the NHS was preferable as budgets and funding were raised as a concern stating that if cuts were made, it would be more likely funding would be cut from programmes such as these. Ensuring the programme was free to the patient was seen as a priority in order to help with adherence to such a programme.

*“I mean, if there's provision for a charity or if there's provision for any money by the NHS that's going to be ... so if it's still free to the patient, I'd rather tap into it because you know, we're in austerity at the moment ...”* (URO)

*“Yeah, I do. I just think within the NHS, the thing to go when budgets are slashed is something like this, like you were talking about mental health services.”* (URO)

- - **Cost-Effectiveness**

HCPs suggested that because of the multi-factorial benefits of exercise, such programmes might be cost-effective given the cost savings that might impact on a range of conditions the NHS has to treat.

*“And it's not just going to help with fatigue, it's going to help psychologically. So perhaps referrals onto our clinical psychologist which probably costs a fortune cos they'll come down. So you know, you drop clinic appointments with CNSs, we won't have to perhaps see them so often because they'll feel better about themselves maybe; it'll reduce phone calls - we get a loads of patient phone calls, you know, just needing a bit of reassurance over the phone.”* (CNS)

*“is there a number of measures that you can use, cos we know that there's evidence of that reduces admissions and we know that you can evidence it, you can really improve quality of life and actually generally you end up with a really good patient experience as well. So you've been able to build the case for that ...”* (CCG)

**Barriers**

**Health Professional Barriers**

Overall, it was initially thought that there may not be a lot of resistance from health care professionals in regards to practically implementing an integrated exercise programme, but possible a lack of engagement due to other factors. A few views on potential barriers were raised.

- **Referral Process**

Having a complicated, time-consuming referral process was suggested to be a barrier for referring men, being able to have simple inclusion criteria, where the men could be assessed by another individual was deemed to be most appropriate.

*“The classic example is, you know, if we refer somebody for an MRI scan, there's a questionnaire we're supposed to complete with the patient - and that is a real headache - um, so .... you know, what you want is seamless and you just want ... you know, as long as I've got 2 arms and 2 legs and I can walk for 5 minutes ... I don't know, I think that's the best you're going to get in clinic ...”* (URO)

- **Resources**

Having an adequately staffed workforce and enough time was a concern of the consultants, addressing lifestyle advices was considered something that maybe is not something that is driven as much as it should be but being over-stretched and needing the time and effort to approach these subjects makes it more difficult. Also, an issue of the increase of health care professional’s role carrying out administrative tasks could cause issues when implementing such a programme.

*“So, as much as I think they would like to get engaged with this I suspect you're going to get 'we haven't got the time to.”* (URO)

*“Yeah, I think probably just having extra staff because, you know, setting things up like this and getting it running, it takes time, and unless you have the workforce ... I'd say the workforce is really important.”* (PHY)

*“Yes. Huge responsibility for the CNS's to take it on board because they might be the only health professionals, together with the consultants, the doctors, that actually have much input with the men in the early stages, but also for the AHP's and people on the ward, if patients do come in for any inpatient treatment, just awareness all round.”* (PHY) *“What is lacking is ... the issue is a lot of administrative tasks come out which aren't ... you don't need a health professional to do them, but that actually does do them because there usually isn't any extra funding for somebody to take that on, and I find that that's a real need now.”* (PHY)

- **Competencies**

Having the appropriate level of staff, with the correct competencies available to run the intervention and assess the patients was mentioned. This barrier had previously created an issue in exercise programmes in other patient groups, when level 4 cancer rehabilitation workers were unable to assess complex patients and there was a need for a physiotherapist. So ensuring that the programme was overseen by a physiotherapist or somebody who was able to assess and work with the needs of complex patients would be necessary. Also, ensuring the health professionals referring onto such a programme were confident in the programme.

*“Fear. I think fear on both sides, I think this blame claim life that we're living in now, I don't think that does the doctors much justice because they don't feel like they've got any support behind them, but that's a whole different ball game isn't it”* (ES)

*“Yeah, them being able to kind of make decisions about more complex patients ... because the way they have to work, they have to work within set protocols and criteria.”* (PHY)

- **Traditional Values**

Traditional values in oncology do not include the integration of exercise training in standard treatment. Such therapies are also not part of the standard medical curriculum that most oncologists and nurses undertake in their first degree.

*“So it's just breaking down them traditional values that some of the older consultants and nurses can have … But ... so that's probably one of the biggest barriers is just breaking down that sort of view point from there … Yeah, they're unaware that exercise is a valid form of rehab.”* (ES)

*“I mean for some people, the idea to put 80-year old people on treadmills is close to torture ... and for other people it is something that they say yes, very very good, it improves everything, it is good.”* (GP)

*“You may get certain, you know, some doctors who just maybe don't see the value of it.”* (CNS)

**Solutions**

- **Evidence Base**

A number of potential barriers were identified, having a good, solid evidence base was deemed to be the most important factor to overcome resistance and embed such a programme into the cancer care pathway. Ensuring that this evidence base is shared amongst the teams was suggested to be crucial and being key to have health care professionals engage in the programme.

*"I mean I think there's evidence that we all should be taking exercise, especially those who are on hormonal treatment so I don't think anyone should criticise that."* (URO)

*“So they can't really say no unless they disagree with the pathway, can they? And I can't see that they would if it's in the NICE guidance ...”* (CNS)

*“I think a lot of doctors want to see evidence, it's like for medication or whatever, but they want to see the research and evidence for how it helps patients.”* (CNS)

- **Specialist further training**

Training staff on the benefits of exercise was suggested to help overcome individuals who were sceptical to the concept of exercise for this patient group. Also to give the nurses the tools to bring up the topic of exercise to the patients.

*“It was also giving the nurses a little bit of knowledge around exercise for those who don't exercise, so it was a bit of teaching on both sides really.”* (ES)

**Patient Barriers**

There was a number of concerns expressed regarding patients participating in a structured exercise programme.

- **Impact of treatment**

The impact of hormone therapy was suggested to be a potential barrier, as it effects men to varying degrees, some men may struggle to partake in exercise because they are fatigued from the treatment. However, individuals who may not have experienced symptoms yet and some individuals who are still working, may also be less likely to engage in exercise.

*“But someone who's starting out from the outset who doesn't have the symptoms and who said ... and you know if they're still working and they've got a busy schedule ... do you know what I mean? I think probably their adherence would be less cos they'll say well no, actually I'm fine at the moment.”* (ES)

*“The reasons they might drop out, like I say, is because they've come unwell or sometimes if they've got a lengthy course of radiotherapy, they just find it overwhelming trying to do that at the same time, things like that, so they might start again once they've finished radiotherapy.”* (PHY)

- **Information Giving**

It was suggested that linking management of side effects to exercise may prove to be an issue to some men, the consultants were concerned the link may not be clear to the patients about the benefits of exercise in connection to their treatment. Therefore concise information about the benefits and programme in question would be essential, to ensure the patients understand the importance of keeping active. Also giving information in a way that is easily accessible to the men, in forms of leaflets etc., maybe with the use of IT versions. In order for the patients to be able to understand the benefits of exercise as current websites such as the department of health are not very patient user friendly. It was also expressed that it was important to acknowledge that exercise may be difficult for certain patient’s dependent upon their cancer care pathway and may not be relevant to every patient, even though there was a general consensus, it should be offered as a treatment. Therefore, there may be an issue of men feeling like a ‘failure’ if they can’t or don’t want to exercise.

*“So I guess that's perhaps something that patients, I guess, may think well it perhaps doesn't quite make sense.”* (URO)

*“They would pop in a tablet thinking it would do a job, but they wouldn't do 15 minutes of exercise thinking it will benefit them.”* (ONC)

*“I mean it's not going to be right for everybody, some people can't do it and I think there's a balance between telling a man he's got to exercise, because some men just don't want to exercise, and they might feel like failures if they're not exercising and they might feel bad about it, but I think it should go hand-in-hand with the ADT.”* (URO)

- **Worries and concerns**

Individual factors were also considered to potentially impact upon adherence to an exercise programme such as other comorbidities, individual coping styles and age. The men’s own concerns about exercise and attending a supervised exercise session was also suggested to maybe hinder uptake. It was expressed that some of the men may not have exercised in a long time and may be concerned about injuries or how they may be perceived in a ‘typical’ gym compared to other men.

*“Well they've all got a bit of arthritis and they've all had, you know, a lot of them have had hip replacements or whatever, they might not be able to do .... depends what they're asked to do, but ... um yeah, it may well limit their activity - their hearts may not be great, their lungs may not be great etc, so yes, that may well limit it.”* (URO)

*“Just because they're not, you know, it's just in our psyche isn't it, women talk and support each other and we're motivated to do that and some men aren't.*” (CNS)

*“Yeah, yeah, especially the older set, like they've not been in a gym since they've stopped playing football at 20 and stuff like that, so they tend to be quite concerned about their own bodies really, about what they can do and looking a little bit stupid in a gym is a major concern, especially for the guys because they'll come in a gym and then they'll see the young guys in the weight room lifting weights and they're just like lifting little weights and stuff, so they get quite concerned about that, about not looking like one of the boys ...”* (ES)

*“I think motivation for exercise is reasonable in a good proportion of patients and I would even say 60-70% would have a good motivation for exercise … but about half of them, despite the motivation, would not be able to do things because other complicating factors arise.”* (ONC)

- **Practicalities**

The location of the exercise programme was suggested to potentially impact upon adherence to an exercise programme. Having a programme that was accessible in the local communities was suggested as preferable as it was expressed that some of the patients don’t like the idea of coming to the hospital, it may be too far for some people to travel and it also may be hard for people to access regularly for exercise.

*“Sometimes having to come back into a hospital environment for some people ... they just have had enough of being in hospital”* (ONC)

*“So that would be a bit of a barrier if it was all centralised to one place.”* (CNS)

*“Transport, money, location, timing, other commitments, fear of being in a group, shame of having this ...”* (CNS)

**Solutions**

- **Necessity**

Being able to see physical health improvements such as weight loss, increased fitness and reduced blood pressure were discussed as a way to keep the men adhering to the exercise programme and increase their exercise behaviour. Therefore having an understanding of the necessity of the exercise programme to help manage their treatment was suggested to be crucial.

*“I think if they are doing better from it .... what you really want is a marker, like a blood test or a urine test that says look, wow, this has gone up, better. I think people respond to rewards don't they.”* (GP)

*“So they become very knowledgeable on what they doing and why they're doing it, so therefore they tend to adhere to it because they know and they can see the difference it's making.”* (ES)

Proposing the exercise as part of their treatment was seen as a way to help improve uptake and also maintain adherence in the men. Having the consultants, initiate the conversation with the patient about an available exercise programme and making exercise sound essential to the men was suggested.

*"I would make it sound like it was part of the treatment, it was packaged in"* (URO)

*“what you need with men I think is that you need the strong advice and direction from the oncologist or the consultant to say this is where you're going, this is your treatment.”* (PHY)

- **Support**

Ensuring the programme is well- supported programme, in a supportive environment which caters for individuals who may be low in confidence was suggested to help with adherence.

*“Patients that are very low in confidence probably do better in the group-based exercise programmes, sort of slightly less formal and patients not having the confidence to walk through a sports centre threshold.”* (PHY)

*“Because it's so supported that they gain confidence and they understand the exercise; like, we're very big on explaining why they're doing exercises so we don't just say right, get on a treadmill and I'll see you later!”* (PHY)

Having the exercise ran in a group was preferable, so the men could gain support and friendships from each other, which would improve adherence. Also having the group, specifically for the men was suggested to be advantageous for adherence rates.

*“Yeah, I think maybe if they were in a group, sort of a group of them together; um, I think if they could feel the benefits of it then they're more likely to carry on doing it and as long as they didn't feel intimidated and they felt well supported, I think that would encourage them to carry on as well.”* (CNS)

**Organisational barriers**

- **Funding**

A lack of money and funding was expressed as being a big issue with the NHS and the NHS current climate. There were concerns expressed about the idea of a 12 month programme due to the issue of funding and provision. Being able to prove that the exercise intervention is cost effective was viewed as a main priority in order to help with the commissioning of the programme. This was suggested to be a possible barrier, as it was proposed to be difficult task. Looking at decreases usages costs as well as an improvement in quality of life, as it was suggested that quality of life may not be good enough evidence due to the financial position the NHS is currently in.

*“I guess the main problem is demonstrating cost-effectiveness isn't it in this day and age, it's soft outcomes ... how do you prove to commissioners that this is something that's worth paying for. I think it's do-able but I think it's challenging … Yeah, you know, whether it decreases primary care usage, whether it decreases hospital usage, whether it improves quality of life - I don't know that improving quality of life is a strong enough outcome measure anymore ...”* (GP)

*“if you could talk to the commissioners and get some evidence to suggest that in the long term it's cost-effective - which it seems like it should be you know, if you keep people fit and well, then they're less likely to be a burden on you later on, then the commissioners would probably pay for that, as a kind of quality of health measure.”* (URO)

*“I think that's a fantastic idea and, in reality, there'd be lots of issues around that; I think if they were carrying on with something for 12 months, there wouldn't be enough funding from either Force charity or the Trust to fund that”* (PHY)

Macmillan and other local charities may be funding current exercise pathways, but there will be a time that CCG's will have to make the decision of whether to continue with funding, money is top of the agenda, especially with this current CCG being in special measures. To be able to prove the programme is cost-effective is key. Good evidence bases also are key, as it cannot be argued with.

*"Absolutely no doubt that it's good for patients, but whether we can actually show some sort of monetary return, and I know there's people at MacMillan working on this, trying to help us and other CCGs to make that argument, you know, that it is cost-effective."* (CCG)

- **Resources**

Having the space and resources to being able to run an exercise programme was suggested to be a possible barrier, as trusts differ in resources and space.

*“I mean, if it's being run as an organisation, you've got to provide a venue and allocate time slots and run it like a clinic or something, then I can imagine huge issues because, you know, at the moment we struggle to get a clinic running.”* (URO)

The potential issues of practically running a group class were raised, for example, in some trusts the amount of men starting on hormone therapy per week is around 1-2, so to build a class may be difficult initially. However, other views were raised suggesting that due to high amount of men initiated on hormone therapy per year, that the NHS would struggle to cater for this amount.

*"I mean, there's evidence that exercise is good for us all isn't it? And everybody knows that, but I'm not sure the NHS can necessarily do exercise regimes for every single person in the country!"* (URO)

*“320 people a year with prostate cancer; about half of those at least will go on hormone treatment and - that's 150-200 a year start on hormone treatment - um, you couldn't have a class with 200 people in it”* (URO)

**Training**

It was suggested that in order for the intervention to be successful, it needs to be supported, therefore training and awareness was identified as a way to tackle this and embed the programme.

- **Practicalities**

Having a face to face training course, that is split up into different sessions, in order to be able to implement new skills in between the sessions was suggested.

*“Honestly I would probably say the best thing to do would be having sessions split up, because then actually if you have homework and you try and implement things between, it's actually far more ... you know you get really motivated by like a 2-day course of something.”* (PHY)

However, in other views deeming that structured training was necessary was suggested, stating that updating health professional’s on the service and progress of the intervention could be discussed at a weekly MDT, so it was ensured that no extra time was taken from the Consultants, but also it was a time where the majority of consultants would be together at one time.

*“Well I think you have to use things that are already in existence locally, because we're all too snowed under to take on extra. So if there's a urology meeting, if the urology department have an MDT meeting or whatever it is where they discuss the cancer patients, that would be the forum.”* (GP)

- **Commitment**

Openness to training was expressed, if it was thought that the end goal would be that the patients were benefitting. Therefore, additional support was welcomed to help utilise their existing skills.

*“if that allowed me to give more information and support to the patients, then you know I'd be more than happy to do that.”* (URO)

*“Yeah, I mean I'm more than happy to have basic training if that empowered me”* (URO)

*“Yeah I think so cos I think we're quite good at recognising where things are changing and where we need to be a little bit better in terms of the support and the strategy we use with our patients, so yeah.”* (CNS)

- **Additional Support**

Additional training was identified to be necessary in the area of behaviour change, communication and goal setting. Training in the referral process, in terms of eligibility to exercise was suggested to be important by the physiotherapists. Also health professionals running the exercise needed to be adequately trained, so it was stated that other health professionals may have an issue in running an exercise intervention.

- **Behaviour Change Support**

Behaviour change support was suggested to be required for the health professionals, with training in goal setting being identified as a need.

*“I probably would need to do a little bit more work into behavioural change to look at really how you guide someone as expertly as possible to make positive health changes, and I guess a big area I would be interested in is sort of motivational interviewing to ensure that it comes from them, and that the programme of the group is set up in the right way that it does deliver change.”* (PHY)

- **Referral Process**

Having a good knowledge of how the referral process worked was suggested to be important and required training.

*“I think it's just having the knowledge isn't it, and the experience to be able to make those judgements and decisions and cos you have to have a good understanding of what the condition is, how it affects people, um, the possible problems that might occur and you have to have really sound knowledge and understanding of all of those things to be able to then prescribe exercise, modify and do it understanding the whole picture of all of those things.”* (PHY)

- **Communication skills**

Having the procedures around the exercise programme taught as part of the common practice was suggested such as initiating a follow up conversation.

*“I think motivational interviewing or having a conversation with people, whether this is an option for them .... Having an infrastructure to run it, that I think is the main issue. Having easy .... not too many communication barriers.”* (GP)

*“So you have to have the ability to do that. I don't think any healthcare professional could do that without, certainly not without training at least.”* (PHY)

*“Definitely, cos then you actually come up with why it's hard, why you probably haven't ... you know, some of these things you do understand and you know already, but why haven't you been utilising it, that extra time you might discuss this, that and the other, why certain conversations or outcome they just fall by the wayside and yeah, I think to really make change for us would be to do it a little bit slower so you actually integrate it into your practice or, you know, whether you're planning to change your groups or just to implement groups gives you better information about how to do that, I'd say.”*

- **Awareness**

Having a great awareness and understanding of the programme was expressed to be important, so for possibly staff to drop into an exercise session to have a first-hand experience was also suggested.

*“Well if it's standard practice that that's all available, then that can be another tick box on the template can't it, there's no problem there. And particularly if they're coming back for an injection and it's administered by the nurse, that can be taught to be a standard part of the injection procedure.”* (GP)

*“Yeah, I guess I'd, you know, like to spend some time with the people that were doing it, doing the exercise with the patients so I could tell the patients more what to expect from it, so I could inform them better?”* (CNS)

**All focus group themes and sub-themes.**

- **Experience of Prostate cancer and hormone therapy**

The experience of being diagnosed with prostate cancer and living with the disease was uniquely individual to the men, which they all acknowledged. However, there were similar trends in terms of the impact of the treatment; side-effects experienced, impact upon quality of life and identity.

- - **Adverse effects of hormone therapy**

The adverse effects of hormone therapy were continually mentioned throughout by the men. A wide range of adverse effects were expressed, these included psychological and physical side effects. The most common side effects reported were weight gain, hot flushes, fatigue, sexual dysfunction, need to urinate more often and changes in their emotional state. These adverse effects are commonly reported by men on this type of treatment.

*“My body shape has changed, I’ve grown breasts.”*

*“Hot flushes do make you tired. It shattered me completely.”*

*“But what you said about going to the toilet, I get that same problem. If I went to bed at 12 o’clock and woke up at 8:00, I’d probably need the toilet four or five times.”*

*“I’m an emotional wreck. I’m not the person that my partner was marrying into.”*

- - **Impact upon Quality of life**

Quality of life was a common issue that was raised with the men and the impact that the treatment had had upon this. The men discussed aspects of their life that had been impacted by the treatment and reported changes to their quality of life in comparison to ‘pre-diagnosis’. Changes to their emotional state, their character and ability to be able to deal with problems were the most recognised:

“*Don’t get me wrong, I have some quality of life, but is it anything like what I was facing two years ago? … It’s absolutely nothing like.”*

Being able to engage in their ‘usual’ activities was enjoyable to the men, however even whilst taking part in other activities, there was still a constant worry about the disease, with this being on their mind, something some found burdensome.

*“It does impact on it, but there’s still a lot left to enjoy. You can still go out, still go the theatre, listen to music, and go for dinner with friends. You know, there is all that, but you are impeded by this horrible thing.”*

The adverse effects were found to impact upon their daily lives and behaviours such as their sleeping patterns. Changes in motivation to partake in their normal activities and ‘do things’ were expressed, with the men’s thoughts about their diagnosis and disease seeming to “dictate” their life, as the men are consumed by these thoughts.

*“Because it is very easy, you know, I’ve been waking up every morning thinking “I don’t feel like getting out of bed today”*

*“I hate it with an absolute passion. It’s because it’s changed my personality so much.”*

The sexual dysfunction suffered from the men due to hormone therapy, was expressed to have an impact upon themselves and also their partners and relationships. Despite their partners being understanding of the disease and treatment, it was something the men did consider that they thought may affect their relationship.

*“Well, no sex for starters ….
But they did say that would happen, didn’t they, and then it has happened.”*

“*I always ask “Are you okay with this?” I have to say that she’s very good, but it’s obviously must have a mental effect on your partner. But it was just from having that injection, it fell off precipitously.”*

- - **Impact upon identity**

Changes to the men physically and psychologically due to the hormone therapy were communicated throughout, these were said to impact upon their individual male identities. Not being able to physically perform usual tasks around the house like DIY was described as ‘annoying’.

Changes physically to their body in terms of weight gain, alterations to their genitalia and a loss of libido, was stated to negatively impact upon the men.

*I’ve lost all that top muscle mass. I’ve started to grow breasts, so you’re putting on lots and lots of body fat.”*

*“It’s like part of you has been erased.”*

- **Coping**

Coping with the diagnosis of prostate cancer, living with the disease and experiencing the effect of hormone therapy differs greatly between individuals. Having adverse side-effects from the hormone therapy was related to increased worry from the men and their daily thoughts being consumed by their concerns.

*“But I did think like that for a while but I didn’t know. I was in the process of renewing my passport when I got told I got prostate cancer, I didn’t bother redoing it because I thought “Oh well, I’m going to be dead this year”. You know this is like, January.”*

Whereas men who had other health conditions which were more symptomatic did not express many worries or concerns about their cancer, as in light of their other health conditions, it is least bothersome.

*“My PSA has gradually gone up and up and up but I’ve had no feeling of - how can I put it - not feeling anxiety about it.”*

The men display different coping mechanisms and styles in relation to their diagnosis, living with prostate cancer and their treatment, due to the differences in treatment effects, symptoms and personal circumstances. Some men having more of an approach style coping – engaging in self-care, discussion of emotions and seeking support, others have adopted more of an avoidant style coping – denial & non-engagement in self-care, this is important to consider as coping styles may impact upon adherence to exercise and lifestyle behaviour.

- - **Approach Coping style**

Engaging in self-care behaviours from diagnosis was different amongst individuals, with men with an approach coping style more likely to engage in self-care. Changes to diet and lifestyle habits were common amongst men who adopted an approach coping style, reporting to have changed their lifestyle habits *“over-night”.* Men with an approach coping style had independently researched information rather than receiving lifestyle related information from health professionals, due to a want for information on lifestyle:

*“You touched on diet, and as soon as I was diagnosed I became vegetarian, I still eat fish, I don’t drink, I don’t have any alcohol at all, I don’t have caffeine, very little salt and no sugar.”*

*“Like you I went online and did as much research as I could around diet and I found that what I do, I believe helps as much as the drugs.”*

Thoughts from the men about the cause of their cancer were briefly expressed, as some men felt that they were able to ‘control’ their cancer by environmental factors, suggested the reason for them making changes to their lifestyle.

*“I think if you’ve created an environment that makes your body react in one way, you’re more than capable of creating an environment that makes your body react in another way.”*

- - **Avoidant Coping style**

Having an avoidant coping style amongst the men resulted in them being less likely to engage in self-care. Being in denial or not wanting to acknowledge their prostate cancer was discussed amongst the men, with some of the men stating that they had not sought any further information or looked into any further support due to them not wanting to acknowledge the disease. Men dealing with the disease themselves without seeking support or supportive programmes was something that was also acknowledged by health care professionals within the prostate cancer care pathway with the small amount of men with prostate cancer accessing support groups compared to other cancer groups.

*“I don’t read about it, I don’t think about it.”*

*“I’ve not spoken really to anybody else, I’ve not tried to sit down and speak to people with cancer. I just thought I’d deal with it.”*

Not discussing their cancer with their partners or others close to them was something some of men chose not to do because they did not want to cause any worry to their partners or their partners did not want to know in depth details about their partner’s cancer.

*“So since I found out, I know it’s serious, but I just don’t tell her because she gets worried too much.”*

*“I’m more or less in the same boat. I don’t associate with anybody except for - on a Wednesday night … But my wife doesn’t want to know what’s going off. She doesn’t want to talk to her about these things.”*

- **Value of Physical Activity**

Physical Activity was identified by the men as a way to keep active, social, and healthy and up taken for enjoyment reasons also. Physical activity is something all the men could relate to and had their own experiences of being physically active throughout their life, placing a large amount of value upon being physically active.

- - **Side-effects of hormone therapy**

A good understanding of the link between exercise and good physical health outcomes, with a few men reporting to have increased their physical activity since their diagnosis after researching into the benefits of exercise on their health.

Exercise prescription schemes such as cardiac rehabilitation had been previously accessed by some of the men who had had heart conditions. Cardiac rehabilitation was found to be enjoyable to the men, due to the group atmosphere and it being individually tailored to them. They had a good understanding of how exercise could help with their heart health but struggled to apply a similar concept to exercise and their prostate cancer.

*“Yes. If it had nothing to do with the heart, but I can’t put the two and two together, the prostate and the exercise.”*

Therefore, more knowledge and information would be needed to ensure the men understand the importance of exercise in the management of their prostate cancer and treatment side-effects. The men highlighted the need for clear, understandable information showing evidence of beneficial effects:

*“If you didn’t have any heart conditions whatsoever and it was just based on prostate, I think if they turned round and said to me that you would need to exercise because of your prostate and it would make you fitter and healthier, I think I would take it up. I really would.”*

Using physical activity as a way to manage the men’s thoughts around their cancer and treatment was suggested to be important and very beneficial to the men. Being able to focus their mind away from their condition and exercising and being amongst others with the same condition and treatment was seen as ‘therapeutic’. The focus groups themselves were found to be supportive to the men, as they were able to learn more about other individual’s experiences, so apply this to an exercise setting was popular amongst men.

*“But exercise and doing things is so important, it’s not something that can be put back by 18 months, it’s something that for different reasons for different people, it’s all intrinsic with the psyche, at a time when we’ve got to fight this horrible disease.”*

- **Adherence**
  - **Factors effecting adherence (barriers)**

Physical activity levels varied amongst individuals, but not being as active as they would like to be was a common issue overall, with all the men stating that they would like to be more active. Having other co-morbidities, joint and muscle pain, worry of injury and a lack of fitness were mentioned as a barrier to being physically active.

*“Yes, we’re wearing out. That’s what it is.”*

*“But I’m wary now of falling over.”*

The impact of hormone therapy and other treatments such as radiation and chemotherapy were stated to have a big impact upon physical ability and fatigue, so the men stated they were motivated to take part in the exercise but sometimes struggled to be able to do the amount of exercise they were used to taking part in prior to treatment. Also the timing of treatment was said to have an impact upon their ability to exercise.

*“It’s not through lack of wanting to, it’s lack of…Radiation stops haemoglobin in your body, so I’ve got half the haemoglobin floating around in my body.”*

*“I’m slightly out of sync, because at the moment I’m having chemotherapy and the chemo tends to have significant effects every three weeks. So I find there’s times where I just don’t do very much at all and the steroids kind of make me put weight on.”*

Exercising and being active in the winter months was suggested as being another barrier by the men, due to finding it hard to get motivated when the weather is cold and it gets darker earlier.

*“So I find it easy to exercise in the summer months, but not so easy in the winter months.”*

*“I go to the gym twice a week and I do miss sometimes, I’ve got to admit, especially if it’s very cold weather, because I don’t like the cold weather at all.”*

When discussing experiences of previous exercise and reasons for adherence, having poor follow up support was mentioned as being the main reason for high dropout rates in programmes such as cardiac rehab.

*“I didn’t get a lot of rehab, to be honest, because I went into chemo.”*

*“Then we were asked “Would you carry on with it at all?” which I did do. Then it gradually faded off.”*

- - **Factors to improve adherence (solutions)**

Exercising in a group setting was seen as a big factor to help improve uptake and adherence rates of an exercise programme amongst the men. This was due to the likelihood of men forming friendships and acquaintances with the other men within the exercise programme, which they highlighted as being a reason to reduce drop-out rates and encourage participation. Whereas if the exercise was not ran in a supervised group, the men thought it would be easy to drop off and it not form part of their lifestyle.

*“In my opinion, it’s better in a group. I know some people like to do it individually, but from personal experience, it’s better in a group, because eventually… When you start, you don’t know each other, but as the time goes by you get to know each other and then you get to know their life story and you get to know… And then it becomes more interesting to go. That’s my opinion anyway.”*

*“I think if you did exercise on your own, you’d drop off eventually. You wouldn’t carry on. You’d do so much”*

Enjoyment was also an important factor help with adherence, ensuring that the men were attending not just as a ‘chore’, but because they actually enjoyed the programme and saw the benefit from it. To have a programme that was individualised and challenging to them was important, also to have a programme that was well supported, due to men having previous experience of cardiac rehab, where lack of support and follow up led to high dropout rates.

*“I think it’s good if you can enjoy it as well, rather than it just being a chore. You’ve got to have a certain amount of enjoyment doing it haven’t you?”*

*“The key thing that would make me engage with it, is if it was individualised to me.
So everybody around this table already does a level of exercise, they just do it differently.”*

Being able to see and measure the improvement in their physical fitness would also be encouraging to the men. Understanding the benefit from attending would help with adherence, for example, seeing that their physical goals being met, but also seeing the benefit participation was having on their physical health, mental health and symptoms illness:

*“And if you think that it has overall, done the body a great of good, I think that will keep you going.”*

*“If somebody got the time to invest in me to say “These are your circumstances and this is what your goals are and this is what you need, and I’ll help you do that”*

- **Patient centred design of exercise schemes**

A consistent interest and want for a supervised exercise programme was expressed by the men. Having access to such a programme was suggested to be an appropriate way to help them manage their side-effects, improve their fitness and health outcomes and lose weight.

*“The exercise is making you feel better, mentally and physically. That’s what it’s all about. Having that drive and going for it.”*

*“No, I’m all for this exercise.”*

- - **Social contact**

Having exercise in a group setting was suggested to help form social circles amongst the men, something that was raised as being very important to the men at the age of retirement, where considerable changes are occurring in their lives. So therefore being able to meet men in similar circumstances would be beneficial at a time they think ‘they need it most’ The initial incentive for the men to attend an exercise programme would be due to their health, but meeting people and forming friendships with other men on the programme would help to maintain their adherence. This was also suggested to help with isolation, something some of the men felt. Also, having the group open to just men with prostate cancer was expressed, with the idea of opening the group up to partners or other cancer groups not being supported.

*“And I think that would happen in the exercise group and one would fire off at another one. And suddenly it would become a friendship group.”*

*“Camaraderie would help because this gentleman has said he feels better because he’s found that this gentleman has had ten years etc.”*

*“I wouldn’t want to with partners. I spend all my time with my partner, I love her to death.”*

- - **Referral Process**

The timing of referral was viewed as “*crucial”.* Views were mixed on when the programme should be offered to the men, however, it was agreed it should not be offered at the end of treatment. Offering a referral onto an exercise programme as soon as treatment is initiated was preferable to some men

*“If exercise is proven to be beneficial to prostate cancer, then it’s beneficial as soon as you diagnose it.”*

Whereas having a period of time to “*digest”* the diagnosis and information, then having access to the programme which would continue whilst they were receiving treatment felt more appropriate to some men:

*“To answer your question, I’d rather on being diagnosed, find out what treatment they’re going to give me. Let me settle down a bit, let me come to terms with where I am and how I’m going to handle it, from both a personal and the family and wider circle of friends.”*

The men felt that it was the consultant’s job to broach the subject of exercise initially, making them aware of the benefits of being active. Having the appropriate written information on the benefits of exercise to support this and information on the programme itself would also be necessary. Further advice would be preferred to be given face to face by the nurse practitioner, with the nurse practitioner being responsible for going through any worries or concerns the men had. Being *“sold”* the exercise by the consultants, and the nurse practitioners would be best placed to provide encouragement:

*“Yes, I agree with that. You have to be sold because you’ve been hit by the fact that you’ve got cancer and you need somebody to actually sell it and say “Look, we think this is going to work for you. I think you should try these exercises”.”*

- - **Information Giving**

The men collectively reported that they had never received any advice regarding lifestyle exercise and diet from the health professionals involved in their prostate cancer care, which they deemed to be “a pity”.

*“I just think it’d be nice to have some guidance though. You know, what to do really, because I’ve not got… I just do things off of my own back.”*

*“The other thing is something that was brought up earlier which I do think is very important. Although this is looking into exercise, I cannot see how you can have, looking into exercise, not to include diet.”*

Due to the initial shock of diagnosis, requesting their health care professional for any supportive programmes was not on their agenda at this time. So therefore, to be given information on this would have been helpful, also helpful to highlight the importance of exercise and management of side-effects of hormone therapy:

*“But it’s still there, it’s still in your head all the time and you don’t think to go and ask for any exercise or anything like that, and it’s not mentioned.”*

Having detailed information about the exercise programme prior to a referral was seen as being key to the men to help them engage into a programme as such. They also expressed a desire for information on projected improvements to their health, condition and the management of their side effects. The men would expect the person responsible for running the exercise to be aware of their health conditions and be able to work around these:

*“I think those conditions would have to be discussed at the beginning. I would say if the people are experts at exercising, then it could work around them.”*

- - **Delivery**

Having the programme run by an “*expert*” or by “*some person who knew what they were doing”* was seen as a high priority to the men, so that they could have a programme individually tailored to them dependent upon their health and any health conditions the men may have. They also thought it may be motivating having the appropriate person running the programme who could help them achieve their individual’s goals, help set new goals with them but also keep a track on their health such as by monitoring their blood pressure and BMI etc.

*“Well I think if it’s an expert taking control of the class, I think they would assess you individually … I think they can be of services. I think the young PTs can tell straight away what you’re capable of and not capable of and they’ll push you to the limit and that bit farther.”*

Having a structured programme run in the local community, twice a week was suggested to be the most appropriate to the men. This would ensure that people would be able to access the programme easily and not have to worry about parking or travelling a long distance twice a week:

*“I think twice is the ideal. I think taking into account, age, I think that it would take some sustaining more than twice a week.”*

If men were unable to attend twice a week due to possible work commitments, the use of a CD or the internet at home was suggested as this may work better, so they could fit it in around their life:

*“I’m a different way. I’m hyperactive, I wouldn’t do that because I’m always busy doing something else. I do it when I can.”*

- - **Emotional Support**

The men viewed their care as very positive but very “clinical” and that there seems to be “gaps” of support regarding their lifestyle and well-being. Whilst they expressed positively that they received good clinical health care, they did however wish for more non-pharmacological support for their well-being, mental health and quality of life and hoped an exercise programme could help to support the men emotionally as well as physically.

*“But it just feels that sometimes you’re falling into gaps where you could be supported.”*

*“In a city with the best consultants and the best healthcare, it would be lacking in advice about other issues.”*

*“But I think it’s too… It’s clinical, clinical, clinical. Nobody sits down and says, “How are you? Are you doing this?”*
