# Supplementary material for: A multi-centre investigation of delivering national guidelines on exercise training for men with advanced prostate cancer undergoing androgen deprivation therapy in the UK NHS
Source: PLoS One. 2018 Jul 5;13(7):e0197606. doi: 10.1371/journal.pone.0197606 (PMC6033384; doi:10.1371/journal.pone.0197606)
Supplement: S7 File — (DOCX) [file pone.0197606.s007.docx]

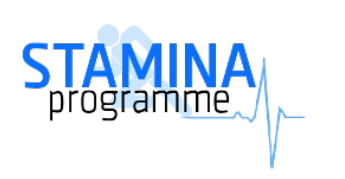
 **S7 File.**

**Site descriptions based on survey and all follow-up data (anonymised)**

## Summary

There is roughly an even split between cancer specific and general long term illness exercise programmes between the sites which we have identified. 9% of the sites identified as having a programme running from the survey; however no further evidence or information could be found online. As a result no further contact could be made via email to obtain more information. Around half of the 47 sites identified had programmes in place which were confirmed as 12 weeks or longer and similarly around half also had some evidence of supervised exercise sessions being in place for patients to access. We can confirm only 17% of sites as having at least two sessions of supervised exercise available to patients and 21% as having exercise specialist involvement in the delivery of the exercise sessions. Only 2 sites were confirmed as being fully integrated into the care pathway for patients and both of these sites had prostate specific exercise programmes. 3 sites in total were identified as having exercise programmes which were prostate cancer specific.

- Number of sites with programmes confirmed 12 weeks or longer (n = 24) 51%
- Number of sites with programmes confirmed supervised exercise (group or 1:1) (n = 25) 53%
- Number of sites with programmes confirmed at least two sessions a week of supervised exercise (n = 8) 17%
- Number of sites with programmes confirmed to be fully integrated (n = 2) 4%
- Number of sites with programmes that have confirmed exercise specialist involvement (n=21) 21%

**Professional roles of respondents** - obtained from the Clinvivo survey data


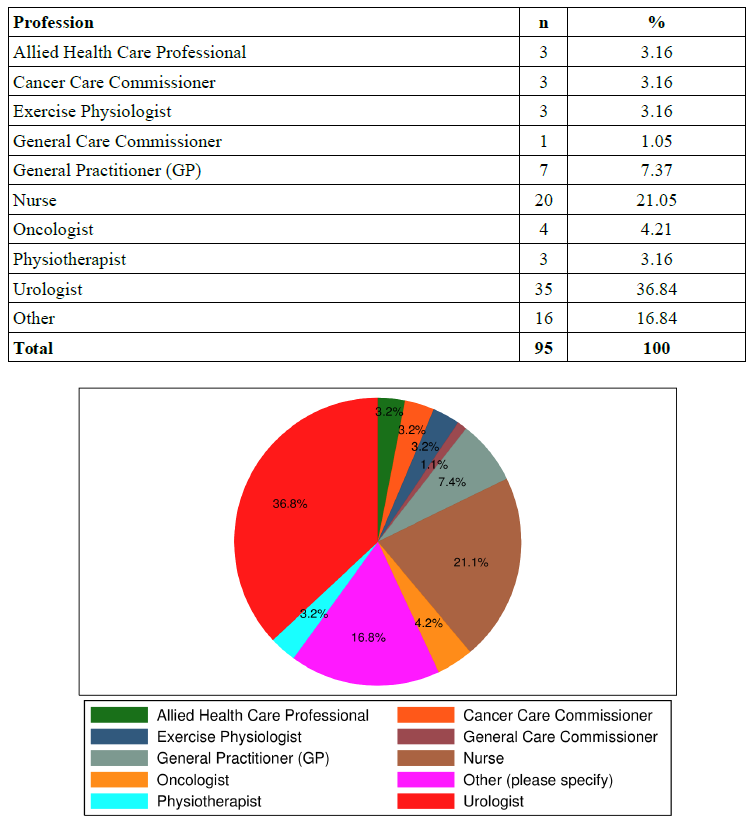


# Sites identified as having exercise programmes in place

**Key: Exercise referral types**

 Long term health conditions in general (n = 19)

Something running but not clear (n = 4)

All cancer types (n = 17)

Prostate cancer specific (n = 3)

Conflicting data (n = 4)

| Type of exercise referral | Professional contacted | Type of programme (1) | Delivery of programme (2) | Additional information | Website(s) |
| --- | --- | --- | --- | --- | --- |
|  | Survey - Get Active referral advisor/ programme manager | - General cancer - Seems to be integrated - A long term programme. | - Referral via CNS - Cancer Rehab (post treatment) | **Emailed – no response** | http://www.getactivebolton.co.uk/specialist-referrals/cancer-rehab/ |
|  | Survey - Allied Health Care Professional  Email - Exercise Referral Officer for BEATS | - For people with a recurring illness/ medical condition. - 12 month programme | - Referral GP, CNS, HCP - Delivery via exercise officers (REPS level 3/4) or by gym instructors - Living with or beyond cancer (must be stable) | Close supervision period for the first 12 weeks with reviews: initial, weeks 6 and 12, months 7 and 12. | http://www.bury.gov.uk/index.aspx?articleid=11710 |
|  | Interview (RT) – GP  Survey -General Practitioner (GP) | - General cancer - based in a gym and studio (Easton, Henbury and Hengrove park leisure centres) mixing aerobic and resistance - No longer a 12 week programme but a 12 session programme for flexibility | - Self-referral and CNS, GP, HCP referral - Cancer Rehab instructors trained to level 4 - Accept all stages of cancer treatment (pre/post and palliative) | **Patients are** given individually tailored programmes. | https://www.bristol.gov.uk/social-care-health/cancer-rehabilitation-exercise |
|  | Survey - Physiotherapist | **Trust led:**   - General cancer?   **Back on Track:**   - General cancer - Group, home or community based dependant on preferences | **Trust Led:**   - Self-referral - 6 week course of Pilates, yoga and tai chi   **Back on Track:**   - Weekly contact - 8 week exercise programme - Post curative treatment (20min consultation with HCP) | **Emailed – no response**  Several LA/ Community/ privately run (walking group) initiatives in the area.  **Back on Track:**  - Individually tailored exercise programme for people who are experiencing cancer related fatigue and cancer related weight loss. | http://survivorship.cancerni.net/services/near-you?tid_1=203&keys=&distance%5Bpostal_code%5D=BT41+2R&distance%5Bsearch_distance%5D=1000&distance%5Bsearch_units%5D=mile&cangen=All  http://belfasttrust-cancerservices.hscni.net/295.htm  http://www.cancerni.net/files/file/Transforming%20Cancer%20Follow%20Up/Jackie%20Gracey%20UU%20Back%20on%20Track.pdf |
|  | Nurse | - General cancer - 12 week programme | - Supervised by instructors trained in exercise rehab - Referral through HCP - For people living with and beyond cancer | Cancer survivorship exercise programme - Includes: Nordic walking, gym sessions, flexibility, Pilates, group circuits and swimming | http://www.bathchronicle.co.uk/Exercise-programme-cancer-sufferers-launched-Bath/story-20044971-detail/story.html |
|  | Nurse | - Prostate cancer specific - 12 week prostate cancer specific programme since Dec 2003. - Integrated - Eight local gyms agreed to take referrals (see Bedford below) | - Delivered by exercise professionals trained to level 4 qualification - Referred by HCP - Men who are about to start ADT (in line with CG175) | **Exercise for life**: Charity funded. Not sure of official programme  **Prostate cancer programme**: All men, with prostate cancer now receive exercise counselling, supported by a written information pack and are offered referral to a 12 week exercise programme | http://www.cuh.org.uk/addenbrookes-hospital/services/cancer-services/support/living-with-and-beyond-cancer/exercise-for-life  https://www.nice.org.uk/sharedlearning/integrating-men-being-treated-for-prostate-cancer-into-exercise-referral-schemes |
|  | General Practitioner (GP) | No data provided/obtainable | No data provided/obtainable | No data provided/obtainable | No data provided/obtainable |
|  | Nurse | - General cancer - Community based (ISE UoD); home based, group exercise or gym programme dependant on patient. | - HCP referrer - Delivered by a CanRehab exercise instructor - Implemented at all stages of cancer care | **Move More**  **Emailed – no response** | http://www.dundee.ac.uk/ise/nonhtdocs/Move%20More%20Dundee%20-%20Referral%20Pathway%20A4%20Poster%20ise.pdf |
|  | **Cancer rehab programme coordinator** | (1) Yes - looks like it based on description of scheme on website. Not fully. *Stepping Out is available to anyone with a cancer diagnosis. We offer 2 exercise classes a week at the local leisure centre, one supported group exercise (circuit based) and one that is a "taster" class in different forms of exercise (i.e Walking football, aqua fit, yoga, qigong).  (2) Yes - walking for health | (3) Referral by GP, consultant or CNS |  | **www.bridportleisure.co/stepping-out-cancer-rehabilitation-referral-scheme.html. (suggested by Dave Franks @ CC)** |
|  | Interview – CNS  Survey - Nurse  Emailed –  Project Manager | **Barts Cancer centre transition programme**   - General cancer - 6 weeks - Community based programme. Works on a group by group basis   **RT - Macmillan exercise prescription programme**   - Run a health and well-being event every 3 months open to all cancers. - General cancer | **Barts Cancer centre transition programme**   - For active and post treatment - Clinical specialists such as an oncologist/s, psychologist, social worker, dietician, and physiotherapist are involved in each 6 week course and this is maintained.   **RT - Macmillan exercise prescription programme**   - 12 weeks, now has a small cost. - HCP refer onto Macmillian social prescribing, who then refer the men themselves onto the necessary programmes. No direct referrals. | The programme has been running for 5 years but there is uncertainty over funding and future of the service. 6 week course with review at 3, 6 and 12 months. Service is audited on an annual basis.  Barts also have a pilot study in prostate cancer rehab to encourage exercise | http://www.bartshealth.nhs.uk/our-services/services-a-z/c/cancer/for-patients/cancer-support/cancer-transitions-programme/  http://prostatecanceruk.org/media/2492258/executive-summary-six-month-pilot.pdf |
|  | Interview – Physio | **RT - Cancer rehab programme**   - General cancer - 8 weeks (twice a week) - General cancer - Community based   **Public Health in Devon**   - Personalised programme; initial one to one consultation with 12-16 week programme. | **Public Health in Devon**   - Referred by HCP - Delivered through qualified instructors | **RT - Cancer rehab programme**  Need for a more structured pathway within urology. Accessed more by females.  GP referral scheme also available.  **Public Health in Devon**  (West, Mid, East Devon; Teignbridge; South Hams) | http://www.devonhealthandwellbeing.org.uk/wp-content/uploads/2012/10/Exercise-Referral-Schemes_Devon.pdf |
|  | Survey - Exercise Physiologist  Emailed – Exercise physiologist | - Both an NHS and privately funded programme. **NHS is only a pilot.** - Not cancer specific but tailored to patient needs. All types of cancer accepted - Private programme is ongoing but **NHS programme is 6 weeks** - Not integrated | - All HCP can refer participants into the programme the majority self-refer. - Exercise physiologist combined with personal trainer deliver. - NHS programme only accept post treatment | The exercises are set but adapted accordingly.  The NHS programme has a pre-assessment week and a post assessment week and follow up telephone calls for a year. The plan is to integrate this into the gym so that the gym can start a maintenance programme too. | http://www.gloucestershireecho.co.uk/Free-fitness-classes-cancer-patients-start/story-26459036-detail/story.html |
|  | Interview – Urologist  Survey - Urologist | No data provided/obtainable | No data provided/obtainable | RT Interview- No formal programme available, diet and exercise information is given to patients via information sheet created by University of Surrey.  Hoping to set up a cancer exercise programme, prostate cancer patients could access within the next few months.  **Two scores of 2 on survey** | http://www.surreyheath.gov.uk/sites/default/files/documents/residents/communities-wellbeing/safer-surrey-heath/SHPrevplanJune2015.pdf |
|  | Urologist | No data provided/obtainable | **No data provided/obtainable** | Survey data - Evidence of specialist involvement Urologist, nurse and gym instructor across all areas (referral and delivery)  Scored 15 |  |
|  | Nurse | No data provided/obtainable | No data provided/obtainable | Survey data - Evidence of specialist involvement Consultant; Nurse; GP; Physiotherapist; Clinical Exercise Physiologist; Gym Instructor across all areas(referral and delivery)  Scored 10 |  |
|  | Interview - UNS | - Local exercise referral scheme for cancer patients. - Exercise available is a wide range – walking groups, exercise classes, swimming etc. - Not integrated |  | Set up and championed by UNS. Small cost, unless patient can’t afford it and the charity will fund them. Around 6% of men with prostate cancer access this. |  |
|  | **Manager** | **(1)** Yes - many. 12wk gym for all people with a cancer diagnosis; 2 x group circuits classes, wide range of abilities and levels, coached swimming, etc. | **(3)** Referral directly by someone within the CIC (not GP's as that is a different scheme) |  |  |
|  | Exercise instructor  Emailed -  Health Development Officer  (Exercise referral scheme) | - Long term illness/ chronic conditions (but have a cancer rehab class) - 12 week programme( broken into two 6 week blocks) - Run through one single centre (Xcel Leisure Complex) - Tailored to the needs of the patient. Sessions are 1:1 and some group. - Not integrated | - Self-referral or referral via a HCP. - Health development officer is responsible for designing the programme for the individual. - Delivery is through an exercise specialist/personal trainer; trained in Cancer rehab Level 4. - Living with or post treatment for caner | Cancer Rehab run once a week cost £3.50 through a village hall.  The programme is ongoing and there are other classes that they can feed in to if they wish. Cost is £90.00.  (Survey) Prostate cancer rehab cannot find evidence online) | http://www.elmbridge.gov.uk/leisure/health/exercisereferral.htm |
|  | **Long term health programme coordinator** | (1) Yes - exercise referral scheme for long term conditions; no end date and can attend up to 5 times per week  (2) Yes - Macmillan Phase III and IV cancer survivors | (3) Referral by any health professional*  * found GP referral takes too long - opened up to physios, oncologists and other HPs to speed up referral process |  |  |
|  | Urologist | - Prostate specific exercise Programme - 12 week exercise and support programme. - 45 minute group circuit and 30 minute support sessions. | - Led by Macmillan Project Officer and CNS - or prostate cancer patients completing treatment - Design of circuit was by a physiotherapist | - Topics included the emotional effects of cancer, continence and erectile dysfunction. - The aim long term is for delivery in a community setting. 2 successful pilots were run. - Consultant Urologist Bill Cross | http://www.leedsth.nhs.uk/a-z-of-services/leeds-cancer-centre/news-events/ |
|  | Exercise Physiologist  Emailed - Responded  Medway Physical Activity Officer | - 12 week referral programme - Long term illness - Patients are set a specific programme around their current circumstances and condition - Conducted through Medways leisure centres - Choice of 1-1 or group.. | - GP or nurse specialist required to refer - Patients living with and beyond cancer can access (including those wanting to get fitter for surgery) - Assessment, induction, 6 and 12 week review. - Supervision can vary - MacMillan sponsored specialist instructor solely dealt with all referrals. Referrals from HCP. | - Though most programmes are gym based, home programmes, water-based activity and Yoga classes are also available. - Exercise will be a part of the cancer care pathway but as of yet Medway Maritime Hospital haven’t rolled this out - Follow-up appointments made at 26 & 52 weeks, but for those having treatment will start the 12weeks rehabilitation once treatment has finished. | http://www.abettermedway.co.uk/getactive/usefulresources/exercisereferral.aspx  file:///C:/Users/Rosaline/Downloads/Presentation%206.1%20Physical%20Activity%20and%20Cancer.pdf |
|  |  | - Prostate cancer specific - 12 week prostate cancer specific programme since Dec 2003. - Integrated - Eight local gyms agreed to take referrals (see Bedford below) | - Delivered by exercise professionals trained to level 4 qualification - Referred by HCP - Men who are about to start ADT (in line with CG175) | **Exercise for life**: Charity funded. Not sure of official programme  **Prostate cancer programme**: All men, with prostate cancer now receive exercise counselling, supported by a written information pack and are offered referral to a 12 week exercise programme | https://www.nice.org.uk/sharedlearning/integrating-men-being-treated-for-prostate-cancer-into-exercise-referral-schemes |
|  | RT interview – exercise specialist  HS interview - Leisure centre  (Survey) Level 4 Exercise specialist | - Long term conditions - Community based exercise referrals - Provide specific, tailored exercise advice and programmes   **RT Interview**   - Free 12-week exercise community programme open to prostate, bowel and breast cancers,   **HS Interview**  (1) Yes - 12 weeks with 8 supervised followed by 4 weeks 'unsupervised' access; attend as many times per week as able. "Can-Move" | - For local GPs and other health agencies   **RT Interview**   - Receive referrals from CNS at Urology department at local hospital via email. Nurse will try 3 times to encourage an exercise referral.   **HS Interview**  (3) Clinical nurse specialist - extending to all HPs from April |  |  |
|  | **Public Health Manager** | (1) Yes - Macmillan Active Manchester Programme | (3) Primary and secondary care pathways / Self-referral / Macmillan Information Library / Cancer support groups | See below |  |
|  | Nurse | **Get Healthy, Get Into Sport:**   - General cancer - built around individual’s needs, one to one or group activity   **The Physical Activity Referral Scheme – PARS:**   - Health screening and advice as well as access to exercise sessions. - Sessions are run across Manchester in local leisure and community centres. - There is a small charge for each activity. - Minimum of 12 weeks | **Get Healthy, Get Into Sport:**   - Living with or beyond cancer - Delivered by team of activity coaches trained in working with cancer patients. - Referrals via HCPs.   **The Physical Activity Referral Scheme – PARS:**   - Led by highly qualified exercise professionals - and prescribes by a physical activity referral officer (up to 12 months) - Referrals via HCPs. |  | http://www.christie.nhs.uk/media/2538/540.pdf  http://www.mhsc.nhs.uk/media/32241/patient%20information%20sheet%20201008.pdf  file:///C:/Users/Rosaline/Downloads/Living_with_and_beyond_cancer_activities.pdf |
|  | Nurse | See above |  |  |  |
|  | Interview – Oncologist/ Urologist  Urologist | **(RG) Interview 2** –   - 12 week exercise referral pathway (attend weekly sessions at a local gym) - General cancer**.**   **Marie Curie Rehabilitation programme:**   - General cancer - Consists of six sessions but further sessions if required. | **(RG) Interview 2**   - Referral to survivorship nurse   **Marie Curie Rehabilitation programme**   - Following an initial assessment by a specialist, a tailored programme will be designed. - HCP referral | **Emailed – no response**  **(RG) Interview 1**- little knowledge of a programme apart from general advice which is given in the context of bone health  **(RG) Interview 2** - **" a specialist nurse who spends 40 minutes with them who talks through all the aspects of their care and then has the time also to then be able to signpost to meaningful interventions that are around…exercise studies here in that group and for that reason, you know, in a research environment”**  See below | https://www.mariecurie.org.uk/globalassets/media/documents/how-we-can-help/hospice-care/our-hospices/newcastle/day-services/newcastle2-0410.pdf |
|  | sport health and fitness specialist | **Live Well**:   - Long term condition exercise referral programme - Carried out in parks, leisure facilities, community centres, schools, open spaces or in places of work. |  | See above | http://activenewcastle.co.uk/health/livewell/ |
|  | Allied Health Care Professional | **National exercise referral scheme**:   - For general chronic illness - The Scheme operates in all 22 local authorities - Runs for 16 consecutive weeks (2 fully supervised group-based sessions each week) - All health data and exercise history is collected and formulated into an activity plan for the individual. - The sessions are usually run in Leisure or Community centres but there are some outdoor opportunities available in most areas. | - GP referral - Exercise professionals operating the Scheme are trained to NVQ level 3 - One to one consultation with an exercise professional | - Follow up consultation at 4 weeks, 16 weeks and 8 months then at 1-year to gage their participation and progress. | http://www.blaenau-gwent.gov.uk/leisure/6302.asp  http://www.wlga.gov.uk/ners |
|  | (Survey) Nurse  Emailed - Vivacity instructor | **Vivacity:**   - Long standing health conditions (classes at reduced rates) - 12 weeks of instructed exercise classes with additional support specific to your condition - Hampton Leisure Center. - There is currently no funding for the scheme and patients pay £2.50 for classes. | - HCP referral, most by GP. - Delivery level four cancer course instructor - Patients can be referred at any stage of the cancer dependent on their treatments. | - “I have just done my level 4 cancer course and will be setting up a new pathway with the macmillen nurses as there is currently nothing specific in the area and no one qualified to teach it.” - 12 weeks of further maintenance classes on a week-by-week basis. After that access to maintenance classes and support groups as you progress. | http://www.vivacity-peterborough.com/sport-healthy-living/specialist-health-services/gp-exercise-referral-scheme/ |
|  | **Centre Coordinator** | - General Cancer - 12 week program. - There are eight Leisure Centre linked to QA Hospital and there is another nine similar schemes across Hampshire. | - HCP referral - Each of the schemes have Exercise Professionals trained in Cancer Rehabilitation Level 4. | **See below** |  |
|  | Interview CNS & Service Manager | RT interview:   - General cancer - 8 week programme - Local university |  | Mainly up taken by breast cancer patients. |  |
|  | (Survey) Cancer Care Commissioner  Emailed -  Macmillan Physical Activity Project Lead for Eastleigh (But works also in the QA Portsmouth): | - 12 week physical activity referral scheme - Long term conditions - Personal exercise programme is designed according to the individual. | - Referral by HCP or self-referral - Can be referred at any point in the cancer pathway - Exercise Professionals trained in Cancer Rehab Level 4 and Behaviour Change interviewing are responsible for delivery | Currently they are working to fully integrate into the patient pathway. | http://www3.hants.gov.uk/exercise-referral-health-checks.pdf  https://www.eastleigh.gov.uk/sport,-countryside,-parks-culture/sport-and-active-lifestyles/healthworks.aspx |
|  | Interview – exercise specialist  (Survey) Exercise referral instructor/ coordinator | **RT Interview**   - 12 week subsidised exercise programme - Open to all health conditions. | - GP referral scheme | - Continual support available after 12 weeks. | http://www.zestcommunity.co.uk/health/exercise-referral-scheme |
|  | Exercise Physiologist |  |  | See above and below |  |
|  | Interview –Urologists (RG)/ physiotherapist (RT)  (Survey) Nurse | RT Interview - Active every day and GP exercise referral schemes available. Nothing specific for prostate cancer. |  | (RG) Interview - no knowledge of a specific programme available for referral for men with PCa. |  |
|  | Public Health | **Fit4Health:**   - Anyone who is inactive or has a longstanding medical condition - 12 week programme - Carried out at local leisure centres. | - HCP referral required; referral officer will arrange a 1-1 consultation where individual needs are assessed - Run by qualified exercise referral instructor | Cannot find evidence of cancer specific pathway online but Active Rotherham and Fit4Health exist. | http://www.placesforpeopleleisure.org/centres/rotherham-leisure-complex/gym-gp-referral |
|  | Interview (RG) – Oncologists  (Survey) Physiotherapist [also had a respondent from the same postcode Urologist who scored 8] | (Survey) Evidence of exercise specialist involvement but little HCP or clinical involvement. Score 12 |  | Cannot find evidence of 12 week programme or prostate specific programme online.  Interviewees (RG) who worked at the RM for six months had no knowledge of an Ex Prog for men with PCa.  Another of the interviewees (RG)**"when I was at the Marsden there were some physios interested in exercise and maintaining exercise and they were doing a study where they gave booklets to patients with exercises in and advice…”**  Personally spoke to Macmillan who informed of a walking group and yoga class for cancer patients at UCLH but no programme exists. 3 other London based interviewees had no knowledge of a programme. | https://www.royalmarsden.nhs.uk/sites/default/files/files_trust/Hot-topics-Cancer-and-Exercise-Programme-Flyer-2016.pdf |
|  | General Practitioner (GP) |  |  | Not clear what is currently on offer  From survey only evidence of physiotherapist involvement. Score 12 | https://moderngov.lambeth.gov.uk/mgConvert2PDF.aspx?ID=1833 |
|  | Exercise Specialist, Exercise on Referral Programme Manager | **Fusion**   - 12 week programme - Long term conditions | - HCP referral required - Accept cancer rehab patients (following treatment). - Carried out by exercise specialists, supervised exercise sessions. | - Telephone follow up at 9 months. - The London Borough of Southwark’s Exercise on Referral programme also exists. | https://www.fusion-lifestyle.com/cms_uploads/file/London_Borough_of_Southwark/Exercise%20on%20Referral%20Southwark.pdf  https://www.fusion-lifestyle.com/contracts/London_Borough_of_Southwark/Community/Health_and_Physical_Activity |
|  | Urologist  Oncologist  Nurse | - General cancer - 12-week programme | - Exercise on referral - Run by physical activity specialists | **Emailed – no response** | http://www.guysandstthomas.nhs.uk/resources/patient-information/therapies/physiotherapy/physical-activity-following-cancer-diagnosis.pdf  http://www.guysandstthomas.nhs.uk/our-services/leips/patients.aspx#na |
|  | Interview RT -CCG | RT Interview –   - General Cancer | - Patients are given a tailored exercise programme, specific for them but no supervision. - Exercise advisor is placed within the hospital. | - Free |  |
|  | **Wellness programme manager** | (1)  - Long term conditions  - 12 weeks gym/circuits and can attend as many times per week as required  - Scheme running in all leisure centres and outdoor facilities city-wide | **(3)** Referral by any health professional | **(2)** Yes - other activities include Nordic walking, swim, cycling, Pilates, walking  --men would pay subsidised rate. |  |
|  | Allied Health Care Professional//Physical Activity Specialsit within a Health promotion Unit working in Primary Care | **Cornwall healthy weight:**   - Long term condition - 12-week programme - Individuals are required to attend once a week, for up to two hours. | - Referral from HCPs. - Trained specialists hold appropriate qualifications recognised by Register of Exercise Professionals. | **Emailed – no response**  Allied health professional working with a unit based in primary care (survey) | https://www.cornwallhealthyweight.org.uk/professional/referral-programmes/ |
|  | Nurse  General Care Commissioner// Development Manager which commissions physical activity, obesity interventions and other healthy Lifestyles, | - Long term conditions - 12 week exercise referral scheme - At least one session of physical activity per week. | - HCP referral - Health & Wellbeing Activators work with patients to plan a programme of appropriate activity - Conducted by qualified instructors. | **Emailed – no response**  Have to pay but sessions are discounted | http://www.wakefield.gov.uk/Documents/sports-leisure/swimming-pools-fitness-centres-golf-courses/Timetables/get-healthy-get-active-web-download.pdf |
|  | Urologist |  |  | Evidence of specialist involvement (survey) Urologist and exercise specialists  Score 14 |  |
|  | Interview - GP |  |  | RT - No specific programme to refer onto – gives general diet and exercise advice. No specific programme available.  Happy to be involved but has concerns about time commitments. |  |
